# Long-term clinical outcomes of oral anticoagulation in the older patients with atrial fibrillation aged ≥80 years: a report from the GLORIA-AF registry phase III

**DOI:** 10.1093/ageing/afaf139

**Published:** 2025-06-04

**Authors:** Hongyu Liu, Yang Chen, Bi Huang, Steven Ho Man Lam, Giulio Francesco Romiti, Yang Liu, Brian Olshansky, Menno Huisman, Kui Hong, Tze-Fan Chao, Gregory Y H Lip

**Affiliations:** Liverpool Centre for Cardiovascular Science at University of Liverpool, Liverpool John Moores University and Liverpool Heart & Chest Hospital, Liverpool L7 8TX, UK; Department of Genetic Medicine, The Second Affiliated Hospital, Jiangxi Medical College, Nanchang University, NO.1, MINDE ROAD, Nanchang, Jiangxi 330006, China; Liverpool Centre for Cardiovascular Science at University of Liverpool, Liverpool John Moores University and Liverpool Heart & Chest Hospital, Liverpool L7 8TX, UK; Liverpool Centre for Cardiovascular Science at University of Liverpool, Liverpool John Moores University and Liverpool Heart & Chest Hospital, Liverpool L7 8TX, UK; Department of Cardiology, The First Affiliated Hospital of Chongqing Medical University, Chongqing, China; Liverpool Centre for Cardiovascular Science at University of Liverpool, Liverpool John Moores University and Liverpool Heart & Chest Hospital, Liverpool L7 8TX, UK; Liverpool Centre for Cardiovascular Science at University of Liverpool, Liverpool John Moores University and Liverpool Heart & Chest Hospital, Liverpool L7 8TX, UK; Department of Translational and Precision Medicine, University of Rome La Sapienza, Rome, Lazio, Italy; Liverpool Centre for Cardiovascular Science at University of Liverpool, Liverpool John Moores University and Liverpool Heart & Chest Hospital, Liverpool L7 8TX, UK; Department of Cardiovascular Medicine, The Second Affiliated Hospital, Jiangxi Medical College, Nanchang University, Nanchang, Jiangxi, China; Jiangxi Provincial Key Laboratory of Molecular Medicine, The Second Affiliated Hospital, Jiangxi Medical College, Nanchang University. Nanchang, Jiangxi, China; Division of Cardiology, The University of Iowa, Iowa City, IO, USA; Department of Medicine – Thrombosis and Hemostasis, Leiden University Medical Center, Leiden, Zuid-Holland, Netherlands; Department of Cardiovascular Medicine, The Second Affiliated Hospital, Jiangxi Medical College, Nanchang University, Nanchang, Jiangxi, China; Jiangxi Provincial Key Laboratory of Molecular Medicine, The Second Affiliated Hospital, Jiangxi Medical College, Nanchang University. Nanchang, Jiangxi, China; Department of Medical Genetics, The Second Affiliated Hospital, Jiangxi Medical College, Nanchang University, Nanchang, Jiangxi, China; Cardiology Department, Taipei Veterans General Hospital, Taipei, Taiwan; Institute of Clinical Medicine, and Cardiovascular Research Center, National Yang Ming Chiao Tung University, Taipei, Taiwan Province, Taiwan; Liverpool Centre for Cardiovascular Science at University of Liverpool, Liverpool John Moores University and Liverpool Heart & Chest Hospital, Liverpool L7 8TX, UK; Department of Clinical Medicine, Aalborg University—Danish Center for Health Services Research, Aalborg, Denmark; Medical University of Bialystok, Bialystok, Poland

**Keywords:** the older, atrial fibrillation, oral anticoagulation, clinical outcome, older people

## Abstract

**Background:**

Older age increases the risk of thromboembolism (TE) and major bleeding in atrial fibrillation (AF) patients, but limited evidence exists regarding the older population (age ≥ 80) especially from different global regions. Data on benefits of oral anticoagulants in these very old individuals are also limited.

**Methods:**

From the prospective, multicenter Global Registry on Long-Term Antithrombotic Treatment in Patients with Atrial Fibrillation registry, we analysed by age all-cause death, cardiovascular death, major adverse cardiovascular events (MACE), TE, major bleeding, stroke, and myocardial infarction (MI) over 3-years follow-up.

**Results:**

Of 7652 patients aged ≥75 years (age 80.1 ± 3.9 years, 47.1% male), 4006 were ≥ 80 years (age 83.4 ± 3.9 years, 43.5% male). After multivariable adjustment, older patients had a higher risk of all-cause death (HR:1.94, 95% CI: 1.67–2.27), cardiovascular death (HR: 2.17, 95% CI: 1.71–2.74), MACE (HR: 1.57, 95% CI: 1.32–1.86), TE (HR: 1.45, 95% CI: 1.14–1.83), major bleeding (HR: 1.30, 95% CI: 1.04–1.63), stroke (HR: 1.38, 95% CI: 1.06–1.80) and MI (HR: 1.59, 95% CI:1.14–2.22). Compared with VKA, NOAC use in patients ≥80 years was associated with lower risks of all-cause death (HR: 0.79, 95% CI: 0.65–0.97), cardiovascular death (HR: 0.70, 95% CI: 0.51–0.96), MACE (HR: 0.72, 95% CI: 0.56–0.92), and major bleeding (HR: 0.66, 95% CI: 0.48–0.92). NOACs were more beneficial than warfarin for mortality, MACE and major bleeding in frail patients. The risk of clinical events associated with older patients was primarily seen in Europe and Asia (*p*_-interaction_ > 0.05), but the effectiveness and safety of NOACs vs. warfarin was consistent across regions.

**Conclusions:**

Older age was independently associated with higher risk of death, major bleeding, TE and MACE. Compared with VKA, NOACs show improved effectiveness and safety in the older and patients with frailty, with similar efficacy across regions and ethnic groups.

## Key points

- Advantages of non-vitamin K antagonist oral anticoagulants (NOACs) over vitamin K antagonists in reducing the risk of bleeding, major adverse cardiovascular events and mortality in atrial fibrillation (AF) patients ≥80 years.
- The most benefit on the mortality, major adverse cardiovascular events and major bleeding of NOACs over vitamin K antagonists were found in the AF patients with frailty.
- Risk of adverse events significantly higher in AF patients ≥80 years than in patients aged 75–79 years.
- High risk of adverse events in patients ≥80 years with AF does not differ significantly by region or ethnic worldwide.

## Introduction

Among the older individuals, atrial fibrillation (AF) is common and contributes to an elevated risk of stroke, systemic embolism, and death [1–3]. Oral anticoagulation (OAC) reduces risk of stroke and all-cause mortality, but introduces a parallel risk of major bleeding, including intracranial haemorrhage [4].

Considering the unique risk–benefit profile of OAC in older patients (age ≥ 80 years), recommendations regarding antithrombotic therapy remains a challenge, particularly due to the limited representation of diverse regional population in clinical trials. This is important, given the reported ethnic differences in stroke and bleeding outcomes [5–8].

Subgroup analyses and post hoc studies of randomized controlled trials (RCTs) have provided some valuable insights for very old AF patients, concerns regarding frailty [9], multimorbidity [10, 11], polypharmacy [12, 13] and high major bleeding risks [14] remain critical considerations in clinical decision-making. Thus, the evidence to guide the anticoagulation therapy in this ‘clinical complex’ population still needs further data. Observational studies and real-world registries offer some insights, but many are small single cohort studies or lack the longitudinal follow-up required to comprehensively evaluate treatment outcomes [15, 16].

Consequently, clinical guidelines for AF management acknowledge the paucity of data for anticoagulation therapy in this subgroup, and emphasize the need for further research [17–19]. Furthermore, differences in drug safety and efficacy between vitamin K antagonists (VKA, e.g. warfarin) and non-vitamin K antagonist oral anticoagulants (NOACs) are particularly relevant in the older AF patients, where age-related physiological changes may alter antithrombotic therapy choices, metabolism and bleeding risks [20, 21].

We aimed to compare the adverse events between AF patients aged 75–79 years and those aged ≥80 and explored differences of clinical outcomes in those treated with VKAs versus NOACs based on age, in a contemporary prospective multicenter global registry.

## Methods

### Study population and data selection

The Global Registry on Long-term Antithrombotic Treatment in Patients with Atrial Fibrillation (GLORIA-AF) is a prospective, global, multicenter registry focusing on newly diagnosed adult patients with non-valvular AF. The study design of GLORIA-AF have been previously described in the previous publication [22], and were registered with ClinicalTrials.gov, NCT01468701 [23]. This study focuses on data from Phase III of GLORIA-AF, covering the period from January 2014 to December 2016, with follow-up of three years and visits conducted at 6, 12, 24, and 36 months.

At baseline, demographic data (age, sex, race, and geographic region), biological characteristics (blood pressure, heart rate, and body mass index [BMI]), lifestyle factors (smoking and alcohol consumption), AF-related symptoms, AF type (paroxysmal, persistent, or permanent), comorbidities, and pharmacotherapies were collected. The CHA_2_DS_2_-VASc score was calculated to assess thromboembolic risk based on the patients’ clinical profile. As GLORIA-AF enrolled globally, data from different regions were explored, that is, Europe, Asia, North America, and Latin America. During the 3-years follow-up, major clinical events were recorded.

### Study groups and clinical outcomes

**Figure 1:**
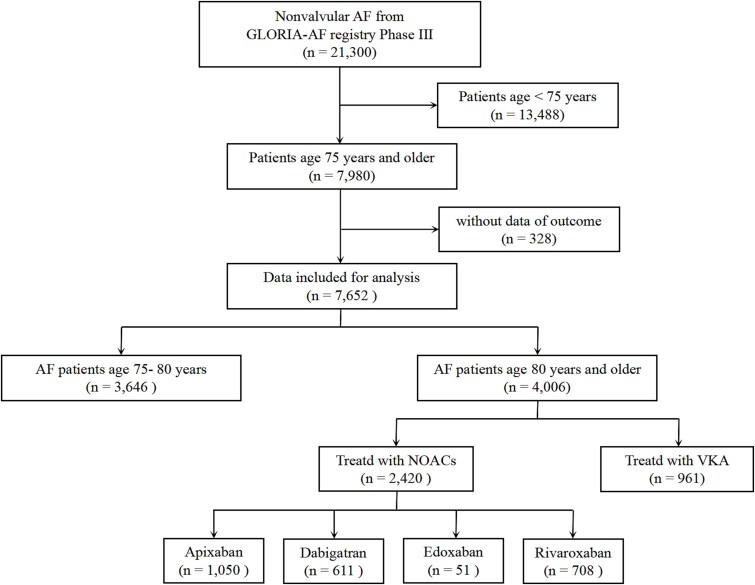
The flowchart of this study. GLORIA-AF, Global Registry on Long-Term Oral Anti-Thrombotic Treatment in Patients with Atrial Fibrillation; AF, atrial fibrillation; VKA, vitamin K antagonists; NOACs, non-vitamin K oral anticoagulants.

This study first compared clinical outcomes between AF patients aged 75–79 years and those aged ≥80 years. Subsequently, the effectiveness and safety of VKA were compared with NOACs in patients aged over 80 years. Regional and ethnic differences were explored in both groups. A flowchart of the study design is shown in Figure 1.

Clinical events were recorded until study withdrawal, death, or the end of the study. The primary clinical outcomes for this analysis included all-cause death, cardiovascular death (CV death), major adverse cardiovascular events (MACE), thromboembolism (TE), major bleeding, stroke, and myocardial infarction (MI). Major bleeding was considered life-threatening bleeding, symptomatic bleeding in critical organs, or a haemoglobin reduction of >20 g/L or requiring ≥2 units of blood transfusion. MACE was defined as a composite of MI, stroke, and CV death. TE included stroke, transient ischemic attack, or systemic embolism outside the central nervous system.

### Statistical analysis

Continuous variables were presented as mean ± standard deviation or median with interquartile range, depending on distribution, and compared using t-tests or Kruskal-Wallis tests. Categorical variables were expressed as counts and percentages and analysed with Pearson’s chi-square test. Kaplan–Meier curves estimated the cumulative incidence of clinical events, with group differences assessed by the log-rank test. Cox proportional hazards regression models were employed to investigate associations between (i) age ≥ 80 years and clinical outcomes (ii) anticoagulant therapy and clinical outcomes in patients aged ≥80 years, with hazard ratios (HRs) and 95% confidence intervals (CIs). To adjust for potential confounders, four models were constructed. Model 1 was univariable analysis. Model 2 was adjusted for demographic and biological characteristics (age, sex, race, region, blood pressure, heart rate, BMI, smoking, and alcohol consumption). Model 3 was further adjusted for comorbidities and prior adverse events (AF type, hypertension, coronary artery disease, chronic heart failure, diabetes, history of TE, history of major bleeding, chronic obstructive pulmonary disease, cancer, and dementia). Model 4, additionally adjusted for baseline medication use, such as angiotensin-converting enzyme inhibitors (ACEI), angiotensin II receptor blockers (ARB), beta-blockers, statins, antiarrhythmic drugs (AAD), aspirin, and OAC.

Subgroup analyses were stratified by sex (male or female), AF type (paroxysmal, non-paroxysmal). In addition, we grouped frailty according to BMI (≤23, >23 kg/m^2^), multimorbidity and polypharmacy to determine whether clinical outcomes were consistent across various populations. The detailed information of prescription drugs and the definition of multimorbidity and polypharmacy were described in Supplementary Table S1. Subgroup interactions were evaluated using likelihood ratio tests to assess the effect of anticoagulation therapy on clinical outcomes across different subgroups.

A *p*-value <0.05 was considered statistically significant. All statistical analyses were performed using R software, version 4.3.1 (R Core Team 2020, Vienna, Austria).

## Results

In GLORIA-AF phase III, 7980 patients were over age 75. After excluding 328 patients with lack of outcome data, the remaining 7652 patients were analysed, of whom, 3646 (47.6%) were 75–79 years old (mean 76.9 ± 3.9; 51.1% male) and 4006 (52.4%) were ≥ 80 years old (83.4 ± 3.9; 43.5% male). Compared with patients aged 75–79 years, patients ≥80 years old were more likely female (56.5% vs. 44.3%), with lower BMI (26.6 ± 5.2 kg/m^2^ vs. 27.8 ± 5.2 kg/m^2^), but had more comorbidities, including hypertension, heart failure, previous TE and previous bleeding (all *P* < .01). Table 1 shows the baseline characteristics of the study cohort, stratified by age groups.

### Clinical events during follow-up

**Figure 2:**
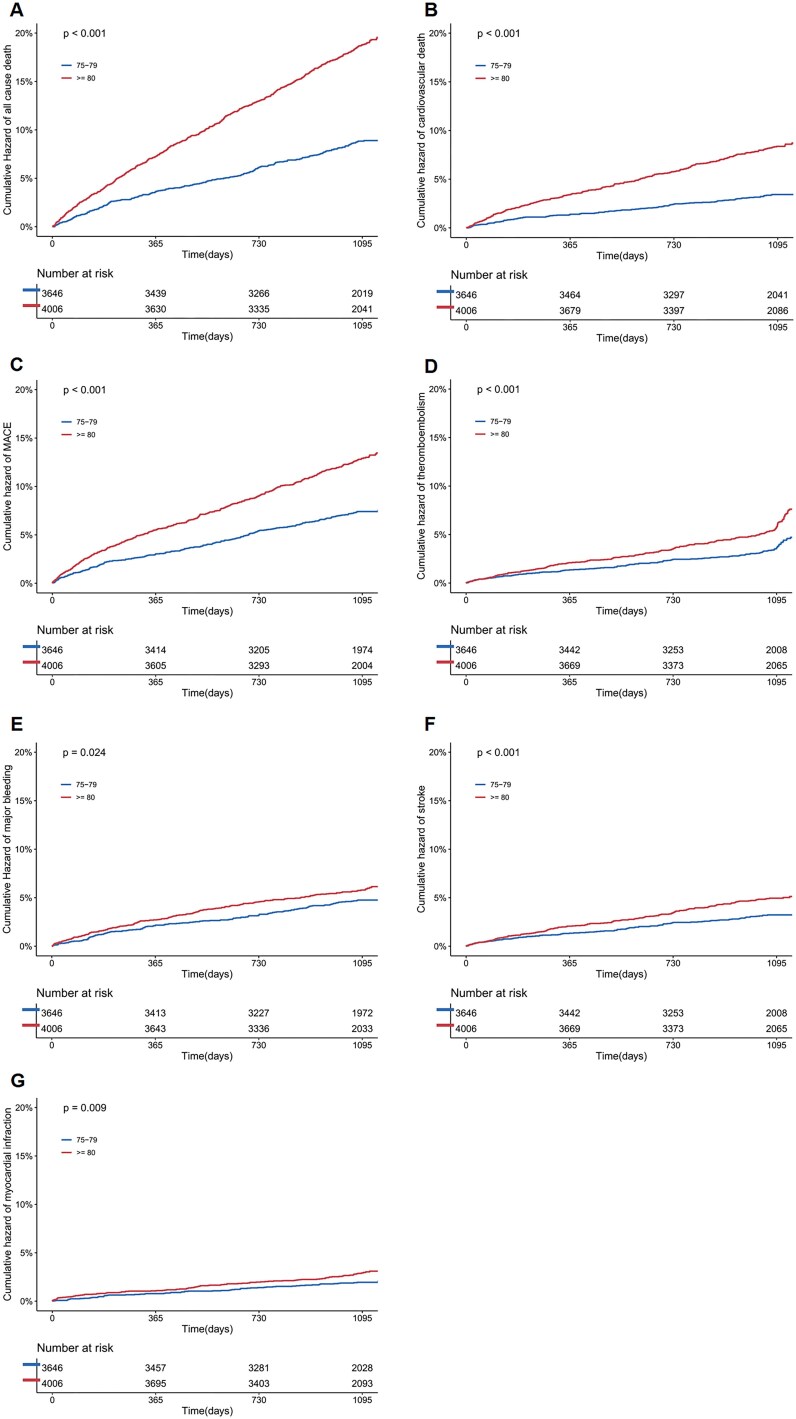
Cumulative event curve in AF patients aged 75–79 years and ≥ 80 years. A-G were all-cause death, cardiovascular death, MACE, thromboembolism, major bleeding, stroke and myocardial fraction, respectively. MACE is a composite included CV death, stroke and MI. Thromboembolism is a composite included transit ischemic attack, stroke and non-CNS arterial embolism. Abbreviation: MACE, major adverse cardiovascular events.

During the 3-years follow-up, 3332 composite clinical events were recorded. Patients ≥80 years old had greater risk all-cause death, CV death, MACE, TE, major bleeding, stroke and MI (all *P* < .05) (Supplementary Table S2). Patients ≥80 years old had higher cumulative hazards of all-cause death (*P* < .001), CV death (*P* < .001), MACE (*P* < .001), TE (*P* < .001), major bleeding (*P* = .024), stroke (*P* < .001), and MI (*P* = .009) (Figure 2).

### Univariable and multivariable analyses

Supplementary Figure S1 shows the associations between age ≥ 80 years and clinical events. The results of multivariable Cox regression model (model 4) were consistent with the univariate Cox regression model and show that AF patients aged ≥80 years were associated with higher risk of all-cause death, CV death, MACE, TE, major bleeding, stroke, and MI.

### Regional and ethnic differences

Among the 7652 patients, 4167 (54.3%) were from Europe, 1082 (14.4%) from Asia, 1763 (22.9%) from North American and 640 (14.4%) from Latin America. Multivariable Cox regression analysis showed that AF patients aged ≥80 years was associated with a higher risk of all-cause death, CV death, TE, and MACE in both Europeans and Asians, while a higher risk of major bleeding was observed only in Europeans. For the regions of North America and Latin America, there were statistically significant higher risks of all-cause death and CV death (Table 2). The increased risks of stroke (HR: 2.71, 95% CI: 1.41–5.24) and MI (HR:3.01, 95% CI:1.07–8.47) in Asia region were consistent with the result of race analysis (Supplementary Table S3). The *p*-values for interaction in all regions and ethnic groups were > 0.05.

**Table 1:** Baseline characteristics of patients with atrial fibrillation aged 75 years and older

Characteristic	Overall (N = 7652)	75–80 years (N = 3646)	≥80 years (N = 4006)	*p* value
Age (years)				<0.001
Mean (SD)	80.1 (3.9)	76.9 (3.9)	83.4 (3.9)	
Median (25%, 75%)	80.0 (77.0, 83.0)	77.0 (76.0, 78.0)	83.0 (81.0, 85.0)	
Sex n, (%)				<0.001
Male	3606 (47.1)	1862 (51.1)	1744 (43.5)	
Female	4046 (52.9)	1634 (44.3)	2262 (56.5)	
Race n, (%)				0.207
White	5642 (73.7)	2669 (73.2)	2973 (74.2)	
Asian	1631 (21.3)	802 (22.0)	829 (20.7)	
Black or Afro-Caribbean	87 (1.1)	48 (1.3)	39 (1.0)	
Arab or Middle East	8 (0.1)	3 (0.1)	5 (0.1)	
Others	284 (3.7)	124 (3.4)	160 (4.0)	
Smoking status n, (%)				<0.001
Never smoked	4882 (63.8)	2248 (61.7)	2634 (65.8)	
Ex-smoker	2258 (29.5)	1106 (30.3)	1152 (58.8)	
Current smoker	512 (6.7)	292 (8.0)	220 (5.4)	
Alcohol status n, (%)				< 0.001
No alcohol	4151 (54.2)	1910 (53.4)	2241 (56.0)	
< 1 drink/week	1836 (24.0)	871 (23.9)	965 (24.1)	
1–7 drinks/week	1324 (17.3)	677 (18.6)	647 (16.1)	
≥8 drinks/week	341 (4.5)	188 (5.1)	153 (3.8)	
BM (kg/m^2^)				< 0.001
Mean (SD)	27.2 (5.2)	27.8 (5.2	26.6 (5.2)	
Median (25%, 75%)	26.6 (23.7, 29.8)	27.1 (24.3, 30.5)	26.1 (23.3, 29.3)	
Typer of AF, n, (%)				0.027
Paroxysmal AF	4059 (53.0)	1961 (53.8)	2098 (52.4)	
Persistent AF	2593 (33.9)	1248 (34.2)	1345 (33.6)	
Permanent AF	1000 (13.1)	437 (12.0)	563 (14.0)	
CHA_2_DS_2_-VASc score				<0.001
Mean (SD)	4.2 (1.2)	4.1 (1.2)	4.3 (1.2)	
Median (25%, 75%)	4.0 (3.0, 5.0)	4.0 (3.0, 5.0)	4.0 (3.0, 5.0)	
Previous disease, n (%)				
Hypertension	5967 (78.0)	2794 (76.6)	3173 (79.2)	0.007
Coronary artery disease	1682 (22.0)	786 (21.8)	896 (22.4)	0.409
Congestive heart failure	1674 (21.9)	748 (20.5)	926 (23.1)	0.006
Diabetes	1697 (22.2)	872 (23.9)	825 (20.6)	< 0.001
TE	1384 (18.1)	548 (15.0)	836 (20.9)	<0.001
Previous bleeding	482 (6.3)	192 (5.3)	290 (7.2)	<0.001
COPD	546 (7.1)	247 (6.8)	299 (7.5)	0.248
Cancer	1057 (13.8)	497 (13.6)	560 (14.0)	0.667
Dementia	99 (1.3)	21 (0.06)	78 (1.9)	<0.001
Multimorbidity	4598 (60.1)	2094 (57.4)	2504 (62.5)	<0.001
Oral anticoagulation, n (%)				0.003
No OAC	710 (14.5)	519 (14.2)	551 (14.9)	
VKA	1757 (24.0)	861 (23.6)	896 (24.3)	
Dabigatran	1221 (16.7)	650 (17.8)	571 (15.5)	
Rivaroxaban	1339 (18.3)	673 (18.5)	666 (18.1)	
Apixaban	1818 (24.8)	864 (23.7)	954 (25.9)	
Edoxaban	126 (1.7)	79 (2.2)	47 (1.3)	
Pharmacotherapy, n (%)				
Any antiplatelet drug	2001 (26.1)	923 (25.3)	1078 (26.9)	0.119
Antiarrhythmic drugs	1536 (20.1)	769 (21.1)	767 (19.1)	0.036
ACEI	2231 (29.2)	1094 (30.0)	1137 (28.4)	0.125
ARB	2037 (26.6)	962 (26.4)	1075 (26.8)	0.676
Beta-blocker	4679 (61.4)	2270 (62.3)	2427 (60.6)	0.139
Statins	3393 (48.3)	1787 (49.0)	1906 (47.6)	0.218
Insulin	305 (4.0)	164 (4.5)	141 (3.5)	0.033
Oral hypoglycemic drugs	1054 (13.8)	544 (14.9)	510 (12.7)	0.006
Diuretic	3297 (43.1)	1455 (39.9)	1842 (46.0)	<0.001
Digoxin	616 (8.0)	249 (6.8)	367 (9.2)	<0.001
Verapamil	79 (1.0)	39 (1.1)	40 (1.0)	0.845
Diltiazem	429 (5.6)	193 (5.3)	236 (5.9)	0.278
PPI	2142 (28.0)	957 (26.2)	1185 (29.6)	0.001
H2-receptor antagonists	218 (2.8)	103 (2.8)	115 (2.9)	0.959
COX2 inhibitor	42 (0.6)	14 (0.4)	28 (0.7)	0.087
SSRI	297 (3.9)	125 (3.4)	172 (4.3)	0.058
Polypharmacy	4594 (60.0)	2187 (60.0)	2407 (60.1)	0.947

Continuous variables were presented with mean (SD) and median (IQR). Category variables were presented with frequency and percentage (%).

Multimorbidity was defined as the presence of more than two comorbidities other than atrial fibrillation.

Polypharmacy was defined as the use of five or more prescription drugs.

Abbreviations: BMI, body mass index; TE, thromboembolism; COPD, chronic obstructive pulmonary disease;

OAC, oral anticoagulants; VKA, vitamin K antagonists; NOACs, Non-vitamin K oral anticoagulants; ACEI, angiotensin-converting enzyme inhibitor; ARB, angiotensin II receptor blocker; PPI, proton-pump inhibitor; SSRI, selective serotonin reuptake inhibitor; SD, standard deviation; IQR, interquartile range.

**Table 2:** HRs (95% CI) for the risk of clinical events comparing AF patients aged 75–79 years with those aged ≥80 years across different regions

	Europe (N = 4167)	Asia (N = 1082)	North America (N = 1763)	Latin America (N = 640)
All cause death				
Crude model, HR (95% CI)	2.35 (1.95, 2.84)	2.34 (1.54, 3.57)	1.69 (1.29, 2.21)	1.77 (1.18, 2.64)
Adjusted model, HR (95% CI)	2.18 (1.75, 2.73)	2.10 (1.32, 3.33)	1.65 (1.24, 2.20)	1.83 (1.21, 2.78)
P for interaction	0.101			
Cardiovascular death				
Crude model, HR (95% CI)	2.65 (1.95, 3.60)	2.98 (1.67, 5.32)	1.88 (1.23, 2.88)	2.09 (1.18, 3.73)
Adjusted model, HR (95% CI)	2.39 (1.70, 3.38)	2.41 (1.30, 4.47)	1.79 (1.17, 2.76)	2.20 (1.20, 4.09)
P for interaction	0.484			
TE				
Crude model, HR (95% CI)	1.64 (1.25, 2.18)	2.89 (1.72, 4.87)	1.27 (0.83, 1.93)	1.14 (0.54, 2.41)
Adjusted model, HR (95% CI)	1.51 (1.08, 2.09)	2.64 (1.51, 4.65)	1.19 (0.76, 1.86)	0.94 (0.39, 2.26)
P for interaction	0.152			
MACE				
Crude model, HR (95% CI)	1.79 (1.44, 2.23)	2.31 (1.54, 3.48)	1.39 (1.03, 1.89)	1.70 (1.03, 2.80)
Adjusted model, HR (95% CI)	1.54 (1.19, 1.99)	2.00 (1.28, 3.12)	1.33 (0.98, 1.84)	1.19 (0.43, 3.35)
P for interaction	0.751			
Major Bleeding				
Crude model, HR (95% CI)	1.43 (1.01, 2.01)	1.84 (0.87, 3.91)	1.04 (0.73, 1.49)	0.95 (0.40, 2.26)
Adjusted model, HR (95% CI)	1.39 (1.01, 1.91)	1.51 (0.63, 3.61)	1.08 (0.74, 1.59)	1.08 (0.42, 2.81)
P for interaction	0.401			
Stroke				
Crude model, HR (95% CI)	1.43 (1.03, 2.00)	2.97 (1.67, 5.29)	1.26 (0.76, 2.07)	1.27 (0.55, 2.94)
Adjusted model, HR (95% CI)	1.14 (0.76, 1.70)	2.71 (1.41, 5.24)	1.12 (0.64, 1.99)	0.98 (0.34, 2.75)
P for interaction	0.929			
MI				
Crude model, HR (95% CI)	1.25 (0.79, 1.96)	3.41 (1.33, 8.71)	1.23 (0.72, 2.10)	2.20 (0.78, 6.20)
Adjusted model, HR (95% CI)	1.26 (0.75, 2.11)	3.01 (1.07, 8.47)	1.20 (0.70, 2.08)	3.85 (0.97, 15.31)
P for interaction	0.531			

Adjusted for age, sex, race, body mass index, systolic blood pressure, smoking/alcohol status, type of AF, hypertension, coronary artery disease, chronic heart failure, diabetes, previous bleeding, TE, COPD, cancer, dementia, oral anticoagulants, ACEI, ARB, arrhythmic drugs, beta-blocker, statin.

MACE is a composite included CV death, stroke and MI.

TE is a composite included transit ischemic attack, stroke and non-CNS arterial embolism.

Abbreviations: AF, atrial fibrillation; CV death, cardiovascular death; MACE, major adverse cardiovascular events; TE, thromboembolism; MI, myocardial fraction; CNS: central nervous system; COPD: chronic obstructive pulmonary disease.

### Subgroup analysis

Supplementary Table S4 shows subgroup analyses indicating no interactions stratified by sex, type of AF for the association between age ≥ 80 and clinical events (all *p*_*-*interaction_ > 0.05). However, in the group with multimorbidity (HR:1.58, 95% CI: 1.18–2.12) and BMI ≤23 kg/m^2^ (HR:1.68, 95% CI:1.02–2.81), patients aged ≥80 years AF patients had higher risk of stroke.

### Analysis of AF patients aged over 80 years

As shown in Supplementary Table S5, 3381 patients (83.4 ± 2.6 years, 43.7% males) were treated with an OAC, of whom 961 were prescribed a VKA and 2420 were prescribed a NOAC. Patients prescribed a NOAC were predominantly female (57.1% vs. 54.0%) and had a greater risk of TE (22.0% vs. 16.5%).

There were 1768 composite clinical events recorded in AF patients aged ≥80 years treated with OAC therapy during the 3-years follow-up (Supplementary Table S6). Kaplan–Meier curves for the clinical outcomes according to OAC therapies are provided in Supplementary Figure S2, A-G. AF patients treated with NOACs had lower cumulative hazards of all-cause death (*P* = .001), CV death (*P* = .019), MACE (*P* = .014), major bleeding (*P* = .012), but the effect of prevent TE (*P* = .560), stroke (*P* = .360) and MI (*P* = .190) was statistically non-significant to VKA.

**Figure 3:**
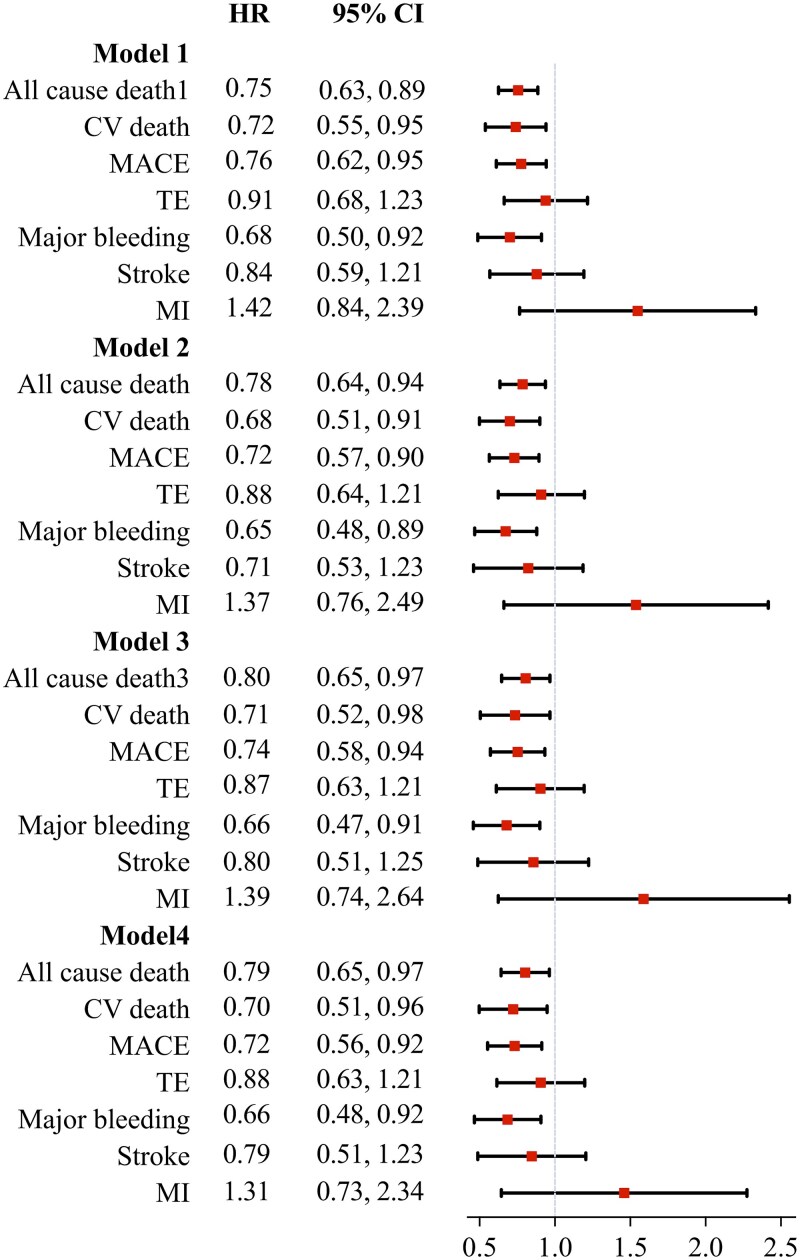
Forest plot of clinical events in AF patients aged ≥80 years by Cox regression analysis comparing treatment group of NOACs with VKA (reference). Model 1: Univariable model. Model 2: Adjusted by age, sex, race, BMI, systolic blood pressure, smoking/alcohol status. Model 3: Adjusted by age, sex, race, BMI, systolic blood pressure, smoking/alcohol status, type of AF, hypertension, coronary artery disease, chronic heart failure, diabetes, previous bleeding, TE, COPD, cancer, dementia. Model 4: age, sex, race, BMI, systolic blood pressure, smoking/alcohol status, type of AF, hypertension, coronary artery disease, chronic heart failure, diabetes, previous bleeding, TE, COPD, cancer, dementia, ACEI, ARB, arrhythmic drugs, beta-blocker, statin. MACE is a composite included CV death, stroke and MI. TE is a composite included transit ischemic attack, stroke and non-CNS arterial embolism. Abbreviation: AF, atrial fibrillation; VKA, vitamin K antagonists; NOACs, Non-vitamin K oral anticoagulants; HR, hazard ratio; 95% CI, 95% confidence interval; CV death, cardiovascular death; MACE, major adverse cardiovascular events; TE, thromboembolism; COPD, chronic obstructive pulmonary disease; MI, myocardial fraction; CNS, central nervous system; ACEI, angiotensin-converting enzyme inhibitor; ARB, angiotensin II receptor blocker.

After adjustment for age, sex, race, BMI, SBP, smoking/alcohol status, type of AF, comorbidities and pharmacotherapy, multivariate Cox regression models show that patients with NOACs had lower risks of all-cause death (HR: 0.79, 95% CI: 0.65–0.97), CV death (HR: 0.70, 95% CI: 0.51–0.96), MACE (HR: 0.72, 95% CI: 0.56–0.92), and major bleeding (HR: 0.66, 95% CI: 0.48–0.92). The risks of TE, stroke and MI were significantly different in patients treated with NOACs and VKA (Figure 3).

The 3-years cumulative incidence of all-cause death, CV death, MACE and major bleeding in patients treated with apixaban or dabigatran were significantly lower than those treated with VKAs (Supplementary Table S7). After adjusting for multiple variables, patients with apixaban had lower risk of all-cause death (HR:0.64, 95% CI: 0.49–0.82), CV death (HR: 0.58, 95% CI: 0.39–0.86), MACE (HR: 0.72, 95% CI: 0.54–0.96) and major bleeding (HR: 0.61, 95% CI: 0.39–0.91), versus patients treated with VKAs. Patients treated with dabigatran had lower risk of all-cause death (HR:0.67, 95% CI: 0.50–0.91), MACE (HR:0.70, 95% CI: 0.49–0.99). Edoxaban, rivaroxaban and warfarin use were not associated with reduced risk of death, TE or MACE (Supplementary Table S8).

### Regional and ethnic differences

In exploratory analyses, associations were observed between anticoagulation and decreased risk of all-cause death and major bleeding in Europe, while there was a significant interaction of NOACs and reduced risk of CV death vs VKAs across all regions (*p*_-interaction_ = 0.017) (Supplementary Table S9). NOACs showed no significant benefit in relation to whites, Asians and other ethnic groups (Supplementary Table S10). No significant interactions were observed for the outcomes investigated in the adjusted regression model, for sex, type of AF and risk of clinical events (Supplementary Table S11).

### Analysis of AF patients with frailty

Frailty was defined by BMI, multimorbidity and polypharmacy. Subgroup analyses revealed no interaction between age ≥ 80 years and clinical events when stratified by these factors. However, the risk of stroke was higher in low BMI (HR: 1.68, 95% CI: 1.02–2.81) and multimorbidity (HR: 1.58, 95% CI: 1.18–2.12) and polypharmacy (HR: 1.75, 95% CI: 1.13–2.71) groups (Supplementary Table S12).

In patients over 80 years old, NOACs significantly reduced MACE risk in low BMI (HR: 0.72, 95% CI: 0.56–0.91) and multimorbid (HR: 0.61, 95% CI: 0.40–0.94) populations, and lowered bleeding risk in polypharmacy (HR: 0.61, 95% CI: 0.41–0.92). There was also no interaction between the three condition and treatment effect (Supplementary Table S13).

## Discussion

In this analysis from the GLORIA-AF Phase III registry, our principal results are as follows: (i) the older patients with AF ≥80 years old had a higher risk of death, MACE, TE and major bleeding during the long-term follow-up versus those aged 75–79 years; (ii) In patients ≥80 years old treated with OAC, NOACs were associated with significantly lower risk of all-cause death, CV death, MACE, and major bleeding; (iii) clinical outcomes, and effectiveness and safety of NOACs vs. warfarin were broadly consistent in different regions of the world; (iv) NOACs showed superior benefit in terms of mortality, MACE and bleeding risk among AF patients with frailty than warfarin.

Previous studies have highlighted the relationship of age and adverse clinical events in patients with AF, especially for those over age 75 [24, 25]. Older individuals often present with multiple comorbid conditions, such as hypertension, diabetes, coronary artery disease, and heart failure [26, 27]. These comorbidities exacerbate the risk of adverse events and complicate anticoagulation management [28, 29]. For instance, renal dysfunction, which is common in this age group, influences the pharmacokinetics of anticoagulants, necessitating dose adjustments and close monitoring [30]. Frailty is a common characteristic in the older adults, with individuals having an ~40% higher risk of developing AF [31]. A meta-analysis of 1,187,651 patients with AF from 33 studies showed that the combined prevalence of frailty was 39.7% and significantly increased the risk of all-cause mortality, ischemic stroke and bleeding in patients with AF [32].

According to previous studies, the older patients with low BMI and multimorbidity are more susceptible to adverse clinical outcomes, key features of frailty [27, 33, 34]. Furthermore, polypharmacy is strongly associated with frailty, increasing risk of mortality in older patients living with frailty [35, 36]. In this study, frailty was assessed based on BMI, multimorbidity and polypharmacy. Older age independently contributed to adverse events, with an even higher stroke risk observed in patients with low BMI, multimorbidity, and polypharmacy. NOACs were associated with lower mortality, MACE, and major bleeding compared to warfarin, consistent with RCTs showing edoxaban's greater benefit in AF patients living with frailty [37, 38].

The CHA_2_DS_2_-VASc (and CHA_2_DS_2_-VA) score assigns 2 points to individuals aged ≥75 years, reflecting the increased thromboembolic risk in this age group [39, 40]. However, our study highlights that patients aged ≥80 years have a significantly higher risk of TE, bleeding, and MACE compared with those aged 75–79 years. Our results suggest additional risk posed by advancing age over 80 and provide critical insights into the clinical management of anticoagulation in the very old patients. Importantly, recent guidelines for AF management also emphasize the limited evidence base for anticoagulation in patients over 80 years, highlighting the urgent need for more robust data to inform clinical decisions in this demographic group [17]. In AF patients over 65 with frailty, NOACs reduced mortality without increasing gastrointestinal bleeding or haemorrhagic stroke risk [41]. Similar findings were evident from randomized trials. For example, edoxaban showed similar stroke prevention but fewer major haemorrhages than warfarin in adults ≥75 years [42], and ELDERCARE-AF study indicated that very-low-dose of edoxaban reduced stroke risk in older patients with frailty [37]. With rising AF prevalence in aging populations, robust anticoagulation evidence in needed. Observation data in AF patients ≥90 linked NOACs to lower intracranial haemorrhage risk without increased ischemic stroke [43]. Another analysis included 327 AF patients age over 80 years indicated that the incidence of major bleeding with warfarin anticoagulation was 1.9 per 100 patient/years [44]. In our global, prospective, real-world analysis, NOACs were associated with reduced mortality and bleeding events in the older population, with similar benefits observed in patients with frailty.

In this study, although there were some variabilities among NOACs, each was at least as effective as warfarin in the prevention of stroke and TE; dabigatran and apixaban were associated with a reduced risk of death and MACE. The superior outcomes observed with apixaban in our study are consistent with findings from a comparative study from Taiwan that showed that apixaban was associated with reduced mortality and adverse event in AF patients aged ≥85 years [45]. In addition, the incidence of stroke or systemic embolism in patients with AF aged ≥80 taking lower-dose dabigatran or rivaroxaban anticoagulation was similar to warfarin [46].

The risk of bleeding events associated with OAC is higher in older patients with AF [47]. Warfarin has a more pronounced rise in the incidence of bleeding with increasing age [48]. In the ENGAGE AF-TIMI 48 trial, AF patients over age 80 receiving low-dose edoxaban had lower risk of major bleeding events (HR 0.59, 95% CI 0.45, 0.77) compared with warfarin, without an increase in ischemic events (HR 0.93, 95% CI 0.69, 1.27) [49]. For very old AF patients with extremely low body weight, edoxaban 15 mg/day was associated with an increased risk of major bleeding [50]. In the ROCKET-AF trial, more gastrointestinal bleeding events occurred with rivaroxaban versus warfarin [51].

In this study, apixaban significantly reduced the risk of major bleeding compared with warfarin. However, the impact of edoxaban on major bleeding was less pronounced. This might be due to the small sample size of those taking edoxaban. Although the rate of major bleeding was lower in the group of dabigatran than in the warfarin group, the association of dabigatran and a reduction risk of major bleeding did not reach statistical significance after multivariate adjustment, consistent with previous reports suggesting that dabigatran may carry a similar risk of extracranial bleeding in the older patients to warfarin [52].

Our exploratory analysis of regional and ethnic differences showed that the increased risk of adverse events in AF patients aged ≥80 was mainly observed in Europe and Asia, while in North America, only mortality risk was elevated, with no significant differences in thromboembolic or haemorrhagic risks. The benefit of NOACs over VKAs remained consistent across regions and ethnic groups. Differences in age distribution, socioeconomic status, education, and healthcare development likely contribute to variations in AF management and prognosis across regions. Our previous study found that in Asia and North America, the proportion of female AF patient and those <65 not receiving oral anticoagulation was higher compared with Europe [53]. In addition, Asia regions exhibited a lower rate of oral anticoagulation, a higher frequency of anticoagulation interruptions, and a significantly elevated risk of TE and intracranial haemorrhage compared with Europe and North America [8].

Given the aging global population and the increasing prevalence of AF [54], optimizing anticoagulation therapy in patients ≥80 is crucial to reducing the burden of stroke, cardiovascular events, and mortality. Our findings support the preferential use of NOACs over VKAs in this high-risk population, particularly in those with elevated bleeding risks. Also, the management of AF is more than OAC alone, current guidelines have moved towards a holistic or integrated care approach [55], whereby adherence with the ‘Atrial Fibrillation Better Care’ pathway has been associated with improved clinical outcomes [56, 57].

### Strengths and limitations

Our study analysis the real-world data from the GLORIA-AF registry, a large-scale, multinational, prospective cohort, that highlight the importance of evidence-based anticoagulation strategies to improve outcomes, and the NOACs provide greater benefit than VKAs in the older AF patients.

Our study has several limitations. First, due to the higher risk of bleeding in AF patients aged ≥80, some of whom were not treated with oral anticoagulants, we excluded this group of patients when comparing the safety and efficacy of VKA versus NOACs and were unable to compare the differences in clinical events between anticoagulated versus non-anticoagulated therapy. Next, due to limited data, our study only provided a crude assessment of frailty based on BMI and comorbidities. In addition, when subgroup analyses of NOACs were performed, the sample size of edoxaban users was small. Also, the small sample size of patients came from Asian in the regional analysis limited statistical power for incidence rates of clinical events. Finally, we did not correct for the effects of INR and time in the therapeutic range on the outcome events of anticoagulation therapy with warfarin due to lack of relevant data. However, the benefit of NOACs over warfarin may be attenuated if the time in the therapeutic range of warfarin is sufficiently high [58].

## Conclusions

Older age was independently associated with higher risk of death, major bleeding, TE and cardiovascular adverse events. Compared with VKA, NOAC use was associated with improved outcomes including survival, major bleeding and reduced cardiovascular adverse events in the older and AF patients with frailty. There were no major differences based on geographic region or ethnic group.

## Supplementary Material

aa-25-0077-File005_afaf139
