# Supplementary material for: Long-term clinical outcomes of oral anticoagulation in the older patients with atrial fibrillation aged ≥80 years: a report from the GLORIA-AF registry phase III
Source: Age Ageing. 2025 Jun 4;54(6):afaf139. doi: 10.1093/ageing/afaf139 (PMC12133677; doi:10.1093/ageing/afaf139)
Supplement: aa-25-0077-File005_afaf139 [file aa-25-0077-file005_afaf139.docx]

**Long-term Clinical Outcomes of Oral Anticoagulation**

**in the Older Patients with Atrial Fibrillation Aged ≥80 Years:**

**A Report from the GLORIA-AF Registry Phase III**

**Running title:** The Older and Atrial fibrillation

**Supplementary Materials**

[Appendix – List of GLORIA-AF Investigators 3](#_Toc194442534)

[Supplementary Table 1. Deatiled information of prescription drugs 11](#_Toc194442535)

[Supplementary table S2. Three-year cumulative incidence rate of clinical events in AF patients aged in 75-79 years and over 80 years 12](#_Toc194442536)

[Supplemental table S3. HRs (95% CI) for the risk of clinical events comparing AF patients aged 75-79 years with those aged ≥80 years in different race and ethnicity 13](#_Toc194442537)

[Supplementary table S4. Subgroup analysis of the association between clinical events and aged ≥80 years in AF patients 14](#_Toc194442538)

[Supplementary table S5. Baseline characteristics of patients with AF aged ≥80 years 16](#_Toc194442539)

[Supplementary table S6. Clinical events between VKA and NOACs anticoagulant treatment groups in AF patients aged ≥ 80 years 18](#_Toc194442540)

[Supplementary table S7. Clinical events between VKA and different NOAC anticoagulant treatment groups in AF patients aged ≥ 80 years 19](#_Toc194442541)

[Supplementary table S8. HRs (95% CI) of clinical events in AF patients aged ≥ 80 years comparing different type of NOACs with VKA 20](#_Toc194442542)

[Supplementary table S9. Cox regression analysis of the association between clinical events and NOAC in AF patients aged ≥80 years across different regions 22](#_Toc194442543)

[Supplementary table S10. Cox regression analysis of the association between clinical events and NOAC in AF patients aged ≥80 years across different race and ethnicity 24](#_Toc194442544)

[Supplementary table S11. Subgroup analysis of the association between clinical events and NOACs in AF patients aged ≥ 80 years 25](#_Toc194442545)

[Supplementary table S12. The association between clinical events and aged ≥80 years in frail AF patients 27](#_Toc194442546)

[Supplementary table S13. The association between clinical events and NOACs in frail AF patients aged ≥ 80 years 29](#_Toc194442547)

[Supplementary Figure S1. Forest plot of clinical events in AF patients by Cox regression analysis comparing group of aged 75-79 years (reference) and aged ≥80 years 31](#_Toc194442548)

[Supplementary Figure S2. Cumulative event curve in very old AF patients treated with NOACs and VKA. 33](#_Toc194442549)

# Appendix – List of GLORIA-AF Investigators

| Dzifa Wosornu Abban | Bouziane Benhalima | Jei Keon Chae |
| --- | --- | --- |
| Nasser Abdul | Jutta Bergler-Klein | Kathrine Chalamidas |
| Atilio Marcelo Abud | Jean-Baptiste Berneau | Krishnan Challappa |
| Fran Adams | Richard A. Bernstein | Sunil Prakash Chand |
| Srinivas Addala | Percy Berrospi | Harinath Chandrashekar |
| Pedro Adragão | Sergio Berti | Ludovic Chartier |
| Walter Ageno | Andrea Berz | Kausik Chatterjee |
| Rajesh Aggarwal | Elizabeth Best | Carlos Antero Chavez Ayala |
| Sergio Agosti | Paulo Bettencourt | Aamir Cheema |
| Piergiuseppe Agostoni | Robert Betzu | Amjad Cheema |
| Francisco Aguilar | Ravi Bhagwat | Lin Chen |
| Julio Aguilar Linares | Luna Bhatta | Shih-Ann Chen |
| Luis Aguinaga | Francesco Biscione | Jyh Hong Chen |
| Jameel Ahmed | Giovanni Bisignani | Fu-Tien Chiang |
| Allessandro Aiello | Toby Black | Francesco Chiarella |
| Paul Ainsworth | Michael J. Bloch | Lin Chih-Chan |
| Jorge Roberto Aiub | Stephen Bloom | Yong Keun Cho |
| Raed Al-Dallow | Edwin Blumberg | Jong-Il Choi |
| Lisa Alderson | Mario Bo | Dong Ju Choi |
| Jorge Antonio Aldrete Velasco | Ellen Bøhmer | Guy Chouinard |
| Dimitrios Alexopoulos | Andreas Bollmann | Danny Hoi-Fan Chow |
| Fernando Alfonso Manterola | Maria Grazia Bongiorni | Dimitrios Chrysos |
| Pareed Aliyar | Giuseppe Boriani | Galina Chumakova |
| David Alonso | D.J. Boswijk | Eduardo Julián José Roberto Chuquiure Valenzuela |
| Fernando Augusto Alves da Costa | Jochen Bott | Nicoleta Cindea Nica |
| José Amado | Edo Bottacchi | David J. Cislowski |
| Walid Amara | Marica Bracic Kalan | Anthony Clay |
| Mathieu Amelot | Drew Bradman | Piers Clifford |
| Nima Amjadi | Donald Brautigam | Andrew Cohen |
| Fabrizio Ammirati | Nicolas Breton | Michael Cohen |
| Marianna Andrade | P.J.A.M. Brouwers | Serge Cohen |
| Nabil Andrawis | Kevin Browne | Furio Colivicchi |
| Giorgio Annoni | Jordi Bruguera Cortada | Ronan Collins |
| Gerardo Ansalone | A. Bruni | Paolo Colonna |
| M.Kevin Ariani | Claude Brunschwig | Steve Compton |
| Juan Carlos Arias | Hervé Buathier | Derek Connolly |
| Sébastien Armero | Aurélie Buhl | Alberto Conti |
| Chander Arora | John Bullinga | Gabriel Contreras Buenostro |
| Muhammad Shakil Aslam | Jose Walter Cabrera | Gregg Coodley |
| M. Asselman | Alberto Caccavo | Martin Cooper |
| Philippe Audouin | Shanglang Cai | Julian Coronel |
| Charles Augenbraun | Sarah Caine | Giovanni Corso |
| S. Aydin | Leonardo Calò | Juan Cosín Sales |
| Ivaneta Ayryanova | Valeria Calvi | Yves Cottin |
| Emad Aziz | Mauricio Camarillo Sánchez | John Covalesky |
| Luciano Marcelo Backes | Rui Candeias | Aurel Cracan |
| E. Badings | Vincenzo Capuano | Filippo Crea |
| Ermentina Bagni | Alessandro Capucci | Peter Crean |
| Seth H. Baker | Ronald Caputo | James Crenshaw |
| Richard Bala | Tatiana Cárdenas Rizo | Tina Cullen |
| Antonio Baldi | Francisco Cardona | Harald Darius |
| Shigenobu Bando | Francisco Carlos da Costa Darrieux | Patrick Dary |
| Subhash Banerjee | Yan Carlos Duarte Vera | Olivier Dascotte |
| Alan Bank | Antonio Carolei | Ira Dauber |
| Gonzalo Barón Esquivias | Susana Carreño | Vicente Davalos |
| Craig Barr | Paula Carvalho | Ruth Davies |
| Maria Bartlett | Susanna Cary | Gershan Davis |
| Vanja Basic Kes | Gavino Casu | Jean-Marc Davy |
| Giovanni Baula | Claudio Cavallini | Mark Dayer |
| Steffen Behrens | Guillaume Cayla | Marzia De Biasio |
| Alan Bell | Aldo Celentano | Silvana De Bonis |
| Raffaella Benedetti | Tae-Joon Cha | Raffaele De Caterina |
| Juan Benezet Mazuecos | Kwang Soo Cha | Teresiano De Franceschi |
| J.R. de Groot | William French | Christian Hall |
| José De Horta | Keith Friedman | Bing Han |
| Axel De La Briolle | Athena Friese | Seongwook Han |
| Gilberto de la Pena Topete | Ana Gabriela Fruntelata | Joe Hargrove |
| Angelo Amato Vicenzo de Paola | Shigeru Fujii | David Hargroves |
| Weimar de Souza | Stefano Fumagalli | Kenneth B. Harris |
| A. de Veer | Marta Fundamenski | Tetsuya Haruna |
| Luc De Wolf | Yutaka Furukawa | Emil Hayek |
| Eric Decoulx | Matthias Gabelmann | Jeff Healey |
| Sasalu Deepak | Nashwa Gabra | Steven Hearne |
| Pascal Defaye | Niels Gadsbøll | Michael Heffernan |
| Freddy Del-Carpio Munoz | Michel Galinier | Geir Heggelund |
| Diana Delic Brkljacic | Anders Gammelgaard | J.A. Heijmeriks |
| N. Joseph Deumite | Priya Ganeshkumar | Maarten Hemels |
| Silvia Di Legge | Christopher Gans | I. Hendriks |
| Igor Diemberger | Antonio Garcia Quintana | Sam Henein |
| Denise Dietz | Olivier Gartenlaub | Sung-Ho Her |
| Pedro Dionísio | Achille Gaspardone | Paul Hermany |
| Qiang Dong | Conrad Genz | Jorge Eduardo Hernández Del Río |
| Fabio Rossi dos Santos | Frédéric Georger | Yorihiko Higashino |
| Elena Dotcheva | Jean-Louis Georges | Michael Hill |
| Rami Doukky | Steven Georgeson | Tetsuo Hisadome |
| Anthony D'Souza | Evaldas Giedrimas | Eiji Hishida |
| Simon Dubrey | Mariusz Gierba | Etienne Hoffer |
| Xavier Ducrocq | Ignacio Gil Ortega | Matthew Hoghton |
| Dmitry Dupljakov | Eve Gillespie | Kui Hong |
| Mauricio Duque | Alberto Giniger | Suk keun Hong |
| Dipankar Dutta | Michael C. Giudici | Stevie Horbach |
| Nathalie Duvilla | Alexandros Gkotsis | Masataka Horiuchi |
| A. Duygun | Taya V. Glotzer | Yinglong Hou |
| Rainer Dziewas | Joachim Gmehling | Jeff Hsing |
| Charles B. Eaton | Jacek Gniot | Chi-Hung Huang |
| William Eaves | Peter Goethals | David Huckins |
| L.A Ebels-Tuinbeek | Seth Goldbarg | kathy Hughes |
| Clifford Ehrlich | Ronald Goldberg | A. Huizinga |
| Sabine Eichinger-Hasenauer | Britta Goldmann | E.L. Hulsman |
| Steven J. Eisenberg | Sergey Golitsyn | Kuo-Chun Hung |
| Adnan El Jabali | Silvia Gómez | Gyo-Seung Hwang |
| Mahfouz El Shahawy | Juan Gomez Mesa | Margaret Ikpoh |
| Mauro Esteves Hernandes | Vicente Bertomeu Gonzalez | Davide Imberti |
| Ana Etxeberria Izal | Jesus Antonio Gonzalez Hermosillo | Hüseyin Ince |
| Rudolph Evonich III | Víctor Manuel González López | Ciro Indolfi |
| Oksana Evseeva | Hervé Gorka | Shujiro Inoue |
| Andrey Ezhov | Charles Gornick | Didier Irles |
| Raed Fahmy | Diana Gorog | Harukazu Iseki |
| Quan Fang | Venkat Gottipaty | C. Noah Israel |
| Ramin Farsad | Pascal Goube | Bruce Iteld |
| Laurent Fauchier | Ioannis Goudevenos | Venkat Iyer |
| Stefano Favale | Brett Graham | Ewart Jackson-Voyzey |
| Maxime Fayard | G. Stephen Greer | Naseem Jaffrani |
| Jose Luis Fedele | Uwe Gremmler | Frank Jäger |
| Francesco Fedele | Paul G. Grena | Martin James |
| Olga Fedorishina | Martin Grond | Sung-Won Jang |
| Steven R. Fera | Edoardo Gronda | Nicolas Jaramillo |
| Luis Gustavo Gomes Ferreira | Gerian Grönefeld | Nabil Jarmukli |
| Jorge Ferreira | Xiang Gu | Robert J. Jeanfreau |
| Claudio Ferri | Ivett Guadalupe Torres Torres | Ronald D. Jenkins |
| Anna Ferrier | Gabriele Guardigli | Carlos Jerjes Sánchez |
| Hugo Ferro | Carolina Guevara | Javier Jimenez |
| Alexandra Finsen | Alexandre Guignier | Robert Jobe |
| Brian First | Michele Gulizia | Tomas Joen-Jakobsen |
| Stuart Fischer | Michael Gumbley | Nicholas Jones |
| Catarina Fonseca | Albrecht Günther | Jose Carlos Moura Jorge |
| Luísa Fonseca Almeida | Andrew Ha | Bernard Jouve |
| Steven Forman | Georgios Hahalis | Byung Chun Jung |
| Brad Frandsen | Joseph Hakas | Kyung Tae Jung |
| Werner Jung | Torben Larsen | Nolan Mayer |
| Mikhail Kachkovskiy | Karine Lavandier | John McClure |
| Krystallenia Kafkala | Jessica LeBlanc | Terry McCormack |
| Larisa Kalinina | Moon Hyoung Lee | William McGarity |
| Bernd Kallmünzer | Chang-Hoon Lee | Hugh McIntyre |
| Farzan Kamali | John Lehman | Brent McLaurin |
| Takehiro Kamo | Ana Leitão | Feliz Alvaro Medina Palomino |
| Priit Kampus | Nicolas Lellouche | Francesco Melandri |
| Hisham Kashou | Malgorzata Lelonek | Hiroshi Meno |
| Andreas Kastrup | Radoslaw Lenarczyk | Dhananjai Menzies |
| Apostolos Katsivas | T. Lenderink | Marco Mercader |
| Elizabeth Kaufman | Salvador León González | Christian Meyer |
| Kazuya Kawai | Peter Leong-Sit | Beat j. Meyer |
| Kenji Kawajiri | Matthias Leschke | Jacek Miarka |
| John F. Kazmierski | Nicolas Ley | Frank Mibach |
| P Keeling | Zhanquan Li | Dominik Michalski |
| José Francisco Kerr Saraiva | Xiaodong Li | Patrik Michel |
| Galina Ketova | Weihua Li | Rami Mihail Chreih |
| AJIT Singh Khaira | Xiaoming Li | Ghiath Mikdadi |
| Aleksey Khripun | Christhoh Lichy | Milan Mikus |
| Doo-Il Kim | Ira Lieber | Davor Milicic |
| Young Hoon Kim | Ramon Horacio Limon Rodriguez | Constantin Militaru |
| Nam Ho Kim | Hailong Lin | Sedi Minaie |
| Dae Kyeong Kim | Gregory Y. H. Lip | Bogdan Minescu |
| Jeong Su Kim | Feng Liu | Iveta Mintale |
| June Soo Kim | Hengliang Liu | Tristan Mirault |
| Ki Seok Kim | Guillermo Llamas Esperon | Michael J. Mirro |
| Jin bae Kim | Nassip Llerena Navarro | Dinesh Mistry |
| Elena Kinova | Eric Lo | Nicoleta Violeta Miu |
| Alexander Klein | Sergiy Lokshyn | Naomasa Miyamoto |
| James J. Kmetzo | Amador López | Tiziano Moccetti |
| G. Larsen Kneller | José Luís López-Sendón | Akber Mohammed |
| Aleksandar Knezevic | Adalberto Menezes Lorga Filho | Azlisham Mohd Nor |
| Su Mei Angela Koh | Richard S. Lorraine | Michael Mollerus |
| Shunichi Koide | Carlos Alberto Luengas | Giulio Molon |
| Anastasios Kollias | Robert Luke | Sergio Mondillo |
| J.A. Kooistra | Ming Luo | Patrícia Moniz |
| Jay Koons | Steven Lupovitch | Lluis Mont |
| Martin Koschutnik | Philippe Lyrer | Vicente Montagud |
| William J. Kostis | Changsheng Ma | Oscar Montaña |
| Dragan Kovacic | Genshan Ma | Cristina Monti |
| Jacek Kowalczyk | Irene Madariaga | Luciano Moretti |
| Natalya Koziolova | Koji Maeno | Kiyoo Mori |
| Peter Kraft | Dominique Magnin | Andrew Moriarty |
| Johannes A. Kragten | Gustavo Maid | Jacek Morka |
| Mori Krantz | Sumeet K. Mainigi | Luigi Moschini |
| Lars Krause | Konstantinos Makaritsis | Nikitas Moschos |
| B.J. Krenning | Rohit Malhotra | Andreas Mügge |
| F. Krikke | Rickey Manning | Thomas J. Mulhearn |
| Z. Kromhout | Athanasios Manolis | Carmen Muresan |
| Waldemar Krysiak | Helard Andres Manrique Hurtado | Michela Muriago |
| Priya Kumar | Ioannis Mantas | Wlodzimierz Musial |
| Thomas Kümler | Fernando Manzur Jattin | Carl W. Musser |
| Malte Kuniss | Vicky Maqueda | Francesco Musumeci |
| Jen-Yuan Kuo | Niccolo Marchionni | Thuraia Nageh |
| Achim Küppers | Francisco Marin Ortuno | Hidemitsu Nakagawa |
| Karla Kurrelmeyer | Antonio Martín Santana | Yuichiro Nakamura |
| Choong Hwan Kwak | Jorge Martinez | Toru Nakayama |
| Bénédicte Laboulle | Petra Maskova | Gi-Byoung Nam |
| Arthur Labovitz | Norberto Matadamas Hernandez | Michele Nanna |
| Wen Ter Lai | Katsuhiro Matsuda | Indira Natarajan |
| Andy Lam | Tillmann Maurer | Hemal M. Nayak |
| Yat Yin Lam | Ciro Mauro | Stefan Naydenov |
| Fernando Lanas Zanetti | Erik May | Jurica Nazlić |
| Charles Landau | Torben Larsen | Alexandru Cristian Nechita |
| Giancarlo Landini | Karine Lavandier | Libor Nechvatal |
| Estêvão Lanna Figueiredo | Jessica LeBlanc | Sandra Adela Negron |
| James Neiman | Arnold Pinter | Hamdi Sati |
| Fernando Carvalho Neuenschwander | Fausto Pinto | Irina Savelieva |
| David Neves | R. Pisters | Pierre-Jean Scala |
| Anna Neykova | Nediljko Pivac | Peter Schellinger |
| Ricardo Nicolás Miguel | Darko Pocanic | Carlos Scherr |
| George Nijmeh | Cristian Podoleanu | Lisa Schmitz |
| Alexey Nizov | Alessandro Politano | Karl-Heinz Schmitz |
| Rodrigo Noronha Campos | Zdravka Poljakovic | Bettina Schmitz |
| Janko Nossan | Stewart Pollock | Teresa Schnabel |
| Tatiana Novikova | Jose Polo Garcéa | Steffen Schnupp |
| Ewa Nowalany-Kozielska | Holger Poppert | Peter Schoeniger |
| Emmanuel Nsah | Maurizio Porcu | Norbert Schön |
| Juan Carlos Nunez Fragoso | Antonio Pose Reino | Peter Schwimmbeck |
| Svetlana Nurgalieva | Neeraj Prasad | Clare Seamark |
| Dieter Nuyens | Dalton Bertolim Précoma | Greg Searles |
| Ole Nyvad | Alessandro Prelle | Karl-Heinz Seidl |
| Manuel Odin de Los Rios Ibarra | John Prodafikas | Barry Seidman |
| Philip O'Donnell | Konstantin Protasov | Jaroslaw Sek |
| Martin O'Donnell | Maurice Pye | Lakshmanan Sekaran |
| Seil Oh | Zhaohui Qiu | Carlo Serrati |
| Yong Seog Oh | Jean-Michel Quedillac | Neerav Shah |
| Dongjin Oh | Dimitar Raev | Vinay Shah |
| Gilles O'Hara | Carlos Antonio Raffo Grado | Anil Shah |
| Kostas Oikonomou | Sidiqullah Rahimi | Shujahat Shah |
| Claudia Olivares | Arturo Raisaro | Vijay Kumar Sharma |
| Richard Oliver | Bhola Rama | Louise Shaw |
| Rafael Olvera Ruiz | Ricardo Ramos | Khalid H. Sheikh |
| Christoforos Olympios | Maria Ranieri | Naruhito Shimizu |
| Anna omaszuk-Kazberuk | Nuno Raposo | Hideki Shimomura |
| Joaquín Osca Asensi | Eric Rashba | Dong-Gu Shin |
| eena Padayattil jose | Ursula Rauch-Kroehnert | Eun-Seok Shin |
| Francisco Gerardo Padilla Padilla | Ramakota Reddy | Junya Shite |
| Victoria Padilla Rios | Giulia Renda | Gerolamo Sibilio |
| Giuseppe Pajes | Shabbir Reza | Frank Silver |
| A. Shekhar Pandey | Luigi Ria | Iveta Sime |
| Gaetano Paparella | Dimitrios Richter | Tim A. Simmers |
| F Paris | Hans Rickli | Narendra Singh |
| Hyung Wook Park | Werner Rieker | Peter Siostrzonek |
| Jong Sung Park | Tomas Ripolil Vera | Didier Smadja |
| Fragkiskos Parthenakis | Luiz Eduardo Ritt | David W. Smith |
| Enrico Passamonti | Douglas Roberts | Marcelo Snitman |
| Rajesh J. Patel | Ignacio Rodriguez Briones | Dario Sobral Filho |
| Jaydutt Patel | Aldo Edwin Rodriguez Escudero | Hassan Soda |
| Mehool Patel | Carlos Rodríguez Pascual | Carl Sofley |
| Janice Patrick | Mark Roman | Adam Sokal |
| Ricardo Pavón Jimenez | Francesco Romeo | Yannie Soo Oi Yan |
| Analía Paz | E. Ronner | Rodolfo Sotolongo |
| Vittorio Pengo | Jean-Francois Roux | Olga Ferreira de Souza |
| William Pentz | Nadezda Rozkova | Jon Arne Sparby |
| Beatriz Pérez | Miroslav Rubacek | Jindrich Spinar |
| Alma Minerva Pérez Ríos | Frank Rubalcava | David Sprigings |
| Alejandro Pérez-Cabezas | Andrea M. Russo | Alex C. Spyropoulos |
| Richard Perlman | Matthieu Pierre Rutgers | Dimitrios Stakos |
| Viktor Persic | Karin Rybak | Clemens Steinwender |
| Francesco Perticone | Samir Said | Georgios Stergiou |
| Terri K. Peters | Tamotsu Sakamoto | Ian Stiell |
| Sanjiv Petkar | Abraham Salacata | Marcus Stoddard |
| Luis Felipe Pezo | Adrien Salem | Anastas Stoikov |
| Christian Pflücke | Rafael Salguero Bodes | Witold Streb |
| David N. Pham | Marco A. Saltzman | Ioannis Styliadis |
| Roland T. Phillips | Alessandro Salvioni | Guohai Su |
| Stephen Phlaum | Gregorio Sanchez Vallejo | Xi Su |
| Denis Pieters | Marcelo Sanmartín Fernández | Wanda Sudnik |
| Kai Sukles | Alberta L. Warner | Tiziana Tassinari |
| Julien Pineau | Wladmir Faustino Saporito | Ashis Tayal |
| Jens Taggeselle | Kouki Watanabe | Muzahir Tayebjee |
| Yuichiro Takagi | Jeanne Wei | J.M. ten Berg |
| Amrit Pal Singh Takhar | Christian Weimar | Dan Tesloianu |
| Angelika Tamm | Stanislav Weiner | Salem H.K. The |
| Katsumi Tanaka | Renate Weinrich | Dierk Thomas |
| Tanyanan Tanawuttiwat | Ming-Shien Wen | Serge Timsit |
| Sherman Tang | Marcus Wiemer | Tetsuya Tobaru |
| Aylmer Tang | Preben Wiggers | Andrzej R. Tomasik. |
| Giovanni Tarsi | Andreas Wilke | Mikhail Torosoff |
| Emmanuel Touze | Ping Zhang |  |
| Elina Trendafilova | Jun Zhang |  |
| W. Kevin Tsai | Shui Ping Zhao |  |
| Hung Fat Tse | Yujie Zhao |  |
| Hiroshi Tsutsui | Zhichen Zhao |  |
| Tian Ming Tu | Yang Zheng |  |
| Ype Tuininga | Jing Zhou |  |
| Minang Turakhia | Sergio Zimmermann |  |
| Samir Turk | Andrea Zini |  |
| Wayne Turner | Steven Zizzo |  |
| Arnljot Tveit | Wenxia Zong |  |
| Richard Tytus | L Steven Zukerman |  |
| C Valadão |  |  |
| P.F.M.M. van Bergen |  |  |
| Philippe van de Borne |  |  |
| B.J. van den Berg |  |  |
| C van der Zwaan |  |  |
| M. Van Eck |  |  |
| Peter Vanacker |  |  |
| Dimo Vasilev |  |  |
| Vasileios Vasilikos |  |  |
| Maxim Vasilyev |  |  |
| Srikar Veerareddy |  |  |
| Mario Vega Miño |  |  |
| Asok Venkataraman |  |  |
| Paolo Verdecchia |  |  |
| Francesco Versaci |  |  |
| Ernst Günter Vester |  |  |
| Hubert Vial |  |  |
| Jason Victory |  |  |
| Alejandro Villamil |  |  |
| Marc Vincent |  |  |
| Anthony Vlastaris |  |  |
| Jürgen vom Dahl |  |  |
| Kishor Vora |  |  |
| Robert B. Vranian |  |  |
| Paul Wakefield |  |  |
| Ningfu Wang |  |  |
| Mingsheng Wang |  |  |
| Xinhua Wang |  |  |
| Feng Wang |  |  |
| Tian Wang |  |  |
| David Williams |  |  |
| Marcus L. Williams |  |  |
| Bernhard Witzenbichler |  |  |
| Brian Wong |  |  |
| Ka Sing Lawrence Wong |  |  |
| Beata Wozakowska-Kaplon |  |  |
| Shulin Wu |  |  |
| Richard C. Wu |  |  |
| Silke Wunderlich |  |  |
| Nell Wyatt |  |  |
| John (Jack) Wylie |  |  |
| Yong Xu |  |  |
| Xiangdong Xu |  |  |
| Hiroki Yamanoue |  |  |
| Takeshi Yamashita |  |  |
| Ping Yen Bryan Yan |  |  |
| Tianlun Yang |  |  |
| Yoto Yotov |  |  |
| Ralf Zahn |  |  |
| Stuart Zarich |  |  |
| Sergei Zenin |  |  |
| Elisabeth Louise Zeuthen |  |  |
| Huanyi Zhang |  |  |
| Donghui Zhang |  |  |
| Xingwei Zhang |  |  |

# Supplementary Table 1. Detailed information of prescription drugs

| **Category** | **Prescription drugs** |
| --- | --- |
| Oral anticoagulant | Warfarin, Dabigatran, Rivaroxaban, Edoxaban, Apixaban |
| Antiplatelet drug | Aspirin, Clopidogrel, Prasugrel, Dipyridamole, Ticlopodine |
| Statins | Atorvastatin, Rosuvastatin, Simvastatin, Pravastatin, Pitavastatin |
| Diuretics | Furosemide, Torasemide, Bumetanide, Hydrochlorothiazide, Indapamide, Spironolactone, Eplerenone, Amiloride |
| AAD | Amiodarone, Flecainide, Dronedarone, Procainamide, Quinidine, Mexiletine, Propafenone |
| PPI | Omeprazole, Lansoprazole, Pantoprazole |
| H2-receptor antagonists | Cimetidine, Ranitidine, Famotidine |
| Multimorbidity | The presence of two or more medically diagnosed diseases (hypertension, coronary artery disease, chronic heart failure, diabetes, history of TE, history of major bleeding, chronic obstructive pulmonary disease, cancer, and dementia) other than AF in an individual |
| Polypharmacy | The use of 5 or more prescription drugs (OACs, AADs, antiplatelet drugs, ACEI, ARB, beta-blockers, statins, insulin, oral hypoglycemic drugs, diuretics, digoxin, verapamil, diltiazem, proton pump inhibitor, H2-receptor antagonists, COX2 inhibitors and selective serotonin reuptake inhibitor) |

Abbreviations: AAD, antiarrhythmic drugs; PPI, proton-pump inhibitor; OACs, oral anticoagulants; ACEI, angiotensin-converting enzyme inhibitors; ARB, angiotensin II receptor blockers.

# Supplementary table S2. Three-year cumulative incidence rate of clinical events in AF patients aged in 75-79 years and over 80 years

|  | **75-80 years n, (%)** | ≥ **80 years n, (%)** | ***p* value** |
| --- | --- | --- | --- |
| All cause death | 301 (8.3) | 671 (16.7) | <0.001 |
| CV death | 119 (3.3) | 303 (7.6) | <0.001 |
| MACE | 251 (6.9) | 458 (11.4) | <0.001 |
| TE | 148 (4.1) | 252 (6.3) | <0.001 |
| Major bleeding | 156 (4.2) | 213 (5.3) | 0.037 |
| Stroke | 109 (3.0) | 178 (4.4) | 0.001 |
| MI | 67 (1.8) | 106 (2.6) | 0.025 |

MACE is a composite included CV death, stroke and MI.

TE is a composite included transit ischemic attack, stroke and non-CNS arterial embolism.

AF, atrial fibrillation; CV death, cardiovascular death; MACE, major adverse cardiovascular events; TE, thromboembolism; MI, myocardial fraction; CNS: central nervous system.

# Supplemental table S3. HRs (95% CI) for the risk of clinical events comparing AF patients aged 75-79 years with those aged ≥80 years in different race and ethnicity

|  | **White (N = 5,642)** | **Asian (N = 1,087)** | **Others (N = 923)** |
| --- | --- | --- | --- |
| **All cause death** |  |  |  |
| Crude model, HR (95% CI) | **2.07 (1.77, 2.42)** | **2.29 (1.51, 3.45)** | **2.12 (1.18, 3.80)** |
| Adjusted model, HR (95% CI) | **1.94 (1.64, 2.31)** | **1.98 (1.26, 3.11)** | **2.10 (1.44, 3.07)** |
| P for interaction | 0.923 | | |
| **Cardiovascular death** |  |  |  |
| Crude model | **2.26 (1.76, 2.91)** | **2.66 (1.51, 4.70)** | **4.23 (1.94, 9.24)** |
| Adjusted model | **2.11 (1.61, 2.77)** | **2.12 (1.16, 3.85)** | **2.84 (1.59, 5.09)** |
| P for interaction | 0.508 | | |
| **TE** |  |  |  |
| Crude model, HR (95% CI) | **1.41 (1.10, 1.82)** | **2.57 (1.46, 4.52)** | 2.79 (1.39, 5.59) |
| Adjusted model, HR (95% CI) | **1.38 (1.09, 1.75)** | **2.49 (1.48, 4.19)** | 1.59 (0.49, 5.19) |
| P for interaction | 0.104 | | |
| **MACE** |  |  |  |
| Crude model, HR (95% CI) | **1.58 (1.32, 1.89)** | **2.07 (1.39, 3.08)** | **2.64 (1.26, 5.55)** |
| Adjusted model HR (95% CI) | **1.49 (1.22, 1.81)** | **1.82 (1.18, 2.81)** | **2.45 (1.54, 3.90)** |
| P for interaction | 0.215 | | |
| **Major Bleeding** |  |  |  |
| Crude model, HR (95% CI) | **1.28 (1.05, 1.64)** | 1.72 (0.86, 3.47) | 1.01 (0.51, 2.01) |
| Adjusted model HR (95% CI) | **1.22 (1.01, 1.54)** | 1.59 (0.77, 3.31) | 1.43 (0.16, 1.29) |
| P for interaction | 0.761 | | |
| **Stroke** |  |  |  |
| Crude model, HR (95% CI) | 1.23 (0.92, 1.64) | **2.79 (1.56, 4.98)** | 2.36 (1.17, 4.77) |
| Adjusted model HR (95% CI) | 1.13 (0.82, 1.55) | **2.72 (1.41, 5.23)** | 1.76 (0.43, 7.24) |
| P for interaction | 0.091 | | |
| **MI** |  |  |  |
| Crude model | 1.34 (0.95, 1.90) | **3.01 (1.24, 7.30)** | 1.53 (0.53, 4.38) |
| HR (95% CI) | 1.27 (0.88, 1.84) | **2.68 (1.07, 6.74)** | 4.87 (0.71, 33.30) |
| P for interaction | 0.082 | | |

Adjusted for age, sex, race, body mass index, systolic blood pressure, smoking/alcohol status, type of AF, hypertension, coronary artery disease, chronic heart failure, diabetes, previous bleeding, TE, COPD, cancer, dementia, oral anticoagulants, ACEI, ARB, arrhythmic drugs, beta-blocker, statin.

MACE is a composite included CV death, stroke and MI.

TE is a composite included transit ischemic attack, stroke and non-CNS arterial embolism.

Abbreviations: AF, atrial fibrillation; HR, hazard ratio; 95% CI, 95% confidence interval; CV death, cardiovascular death; MACE, major adverse cardiovascular events; TE, thromboembolism; MI, myocardial fraction; CNS, central nervous system; ACEI, angiotensin-converting enzyme inhibitor; ARB, angiotensin II receptor blocker; COPD, chronic obstructive pulmonary disease.

# Supplementary table S4. Subgroup analysis of the association between clinical events and aged ≥80 years in AF patients

|  | **Subgroups** | **HR (95% CI)** | ***P* value** | ***P* for interaction** |
| --- | --- | --- | --- | --- |
| **All cause death** | **Sex** |  |  | 0.708 |
|  | Male (n=3,606) | **1.89 (1.54, 2.33)** | **<0.001** |  |
|  | Female (n=4,046) | **2.01 (1.60, 2.52)** | **<0.001** |  |
|  | **Type of AF** |  |  | 0.161 |
|  | Paroxysmal AF (n=4,059) | **1.79 (1.45, 2.23)** | **< 0.001** |  |
|  | Non-paroxysmal AF (n=3,593) | **2.13 (1.71, 2.67)** | **< 0.001** |  |
| **Cardiovascular death** | **Sex** |  |  | 0.520 |
|  | Male (n=3,606) | **2.02 (1.46, 2.79)** | **<0.001** |  |
|  | Female (n=4,046) | **2.31 (1.64, 3.28)** | **<0.001** |  |
|  | **Type of AF** |  |  | 0.929 |
|  | Paroxysmal AF (n=4,059) | **2.28 (1.61, 3.25)** | **< 0.001** |  |
|  | Non-paroxysmal AF (n=3,593) | **2.11 (1.52, 2.92)** | **< 0.001** |  |
| **TE** | **Sex** |  |  | 0.453 |
|  | Male (n=3,606) | **1.41 (1.01, 1.97)** | **0.045** |  |
|  | Female (n=4,046) | **1.59 (1.16, 2.17)** | **0.003** |  |
|  | **Type of AF** |  |  | 0.591 |
|  | Paroxysmal AF (n=4,059) | **1.38 (1.01, 1.90)** | **0.046** |  |
|  | Non-paroxysmal AF (n=3,593) | **1.51 (1.08, 2.11)** | **0.014** |  |
| **MACE** | **Sex** |  |  | 0.218 |
|  | Male (n=3,606) | **1.40 (1.09, 1.78)** | **0.006** |  |
|  | Female (n=4,046) | **1.73 (1.34, 2.22)** | **<0.001** |  |
|  | **Type of AF** |  |  | 0.723 |
|  | Paroxysmal AF (n=4,059) | **1.17 (1.16, 1.85)** | **0.001** |  |
|  | Non-paroxysmal AF (n=3,593) | **1.74 (1.35, 2.24)** | **< 0.001** |  |
| **Major bleeding** | **Sex** |  |  | 0.445 |
|  | Male (n=3,606) | **1.35 (1.02, 1.83)** | **0.042** |  |
|  | Female (n=4,046) | 1.22 (0.88,1.69) | 0.233 |  |
|  | **Type of AF** |  |  | 0.149 |
|  | Paroxysmal AF (n=4,059) | **1.66 (1.10, 2.47)** | **0.015** |  |
|  | Non-paroxysmal AF (n=3,593) | 1.18 (0.85, 1.57) | 0.261 |  |
| **Stroke** | **Sex** |  |  | 0.560 |
|  | Male (n=3,606) | 1.27 (0.84, 1.92) | 0.259 |  |
|  | Female (n=4,046) | **1.49 (1.03, 2.17)** | **0.033** |  |
|  | **Type of AF** |  |  | 0.795 |
|  | Paroxysmal AF (n=4,059) | **1.52 (1.08, 2.14)** | **0.017** |  |
|  | Non-paroxysmal AF (n=3,593) | **1.53 (1.09, 2.13)** | **0.011** |  |
| **MI** | **Sex** |  |  | 0.052 |
|  | Male (n=3,606) | 1.02 (0.62, 1.59) | 0.977 |  |
|  | Female (n=4,046) | **2.15 (1.29, 3.60)** | **0.003** |  |
|  | **Type of AF** |  |  | 0.067 |
|  | Paroxysmal AF (n=4,059) | **1.22 (0.81, 1.83)** | **0.333** |  |
|  | Non-paroxysmal AF (n=3,593) | 2.71 (1.44, 2.09) | 0.002 |  |

Model was adjusted by age, sex, race, body mass index, systolic blood pressure, smoking/alcohol status, type of AF, hypertension, coronary artery disease, chronic heart failure, diabetes, previous bleeding, TE, COPD, cancer, dementia, oral anticoagulation, ACEI, ARB, arrhythmic drugs, beta-blocker, statin.

MACE is a composite included CV death, stroke and MI.

TE is a composite included transit ischemic attack, stroke and non-CNS arterial embolism.

Abbreviations: AF, atrial fibrillation; HR, hazard ratio; 95% CI, 95% confidence interval; CV death, cardiovascular death; MACE, major adverse cardiovascular events; TE, thromboembolism; MI, myocardial fraction; CNS, central nervous system; ACEI, angiotensin-converting enzyme inhibitor; ARB, angiotensin II receptor blocker; COPD, chronic obstructive pulmonary disease.

# Supplementary table S5. Baseline characteristics of patients with AF aged ≥80 years

| **Characteristic** | **Overall**  **(N = 3,381)** | **VKA**  **(N = 961)** | **NOAC**  **(N = 2,420)** | ***P* value** |
| --- | --- | --- | --- | --- |
| **Age (years)** |  |  |  | 0.478 |
| Mean (SD) | 83.4 (2.6) | 83.4 (2.6) | 83.3 (2.6) |  |
| Median (25%, 75%) | 83.0 (81.0, 85.0) | 83.0 (81.0, 85.0) | 83.0 (81.0, 85.0) |  |
| **Sex n, (%)** |  |  |  | 0.104 |
| Male | 1,479 (43.7) | 442 (46.0) | 1,037 (42.8) |  |
| Female | 1,902 (56.3) | 519 (54.0) | 1,383 (57.1) |  |
| **Race n, (%)** |  |  |  | < 0.001 |
| White | 2,604 (77.0) | 789 (82.1) | 1,815 (75.0) |  |
| Asian | 603 (17.8) | 136 (14.2) | 467 (19.3) |  |
| Black or Afro-Caribbean | 32 (1.0) | 8 (0.8) | 24 (1.0) |  |
| Arab or Middle East | 4 (0.1) | 1 (0.1) | 3 (0.1) |  |
| Others | 138 (4.1) | 27 (2.8) | 111 (4.6) |  |
| **Smoking status n, (%)** |  |  |  | 0.001 |
| Never smoked | 2,202 (65.1) | 641 (66.7) | 1,561 (64.5) |  |
| Ex-smoker | 994 (29.4) | 289 (30.1) | 705 (29.1) |  |
| Current smoker | 185 (5.5) | 31 (3.2) | 154 (6.4) |  |
| **Alcohol status n, (%)** |  |  |  | 0.644 |
| No alcohol | 1,843 (54.5) | 534 (55.6) | 1,309 (54.1) |  |
| < 1 drink/week | 840 (24.9) | 240 (25.0) | 600 (24.8) |  |
| 1-7 drinks/week | 568 (16.8) | 149 (15.5) | 419 (17.3) |  |
| ≥8 drinks/week | 130 (3.8) | 38 (3.9) | 92 (3.8) |  |
| **BMI (kg/m^2^)** |  |  |  | 0.326 |
| Mean (SD) | 26.8 (5.1) | 27.0 (5.1) | 26.8 (5.1) |  |
| Median (25%, 75%) | 26.3 (23.5, 29.5) | 26.4 (23.7, 29.7) | 26.3 (23.4, 29.4) |  |
| **Typer of AF, n, (%)** |  |  |  | < 0.001 |
| Paroxysmal AF | 1,701 (50.3) | 405 (42.1) | 1,296 (53.5) |  |
| Persistent AF | 1,171 (34.6) | 375 (39.0) | 796 (32.9) |  |
| Permanent AF | 509 (15.1 | 181 (18.9) | 328 (13.6) |  |
| **CHA_2_DS_2_-VASc score** |  |  |  | 0.807 |
| Mean (SD) | 4.3 (1.3) | 4.3 (1.3) | 4.3 (1.3) |  |
| Median (25%, 75%) | 4.0 (3.0, 5.0) | 4.0 (3.0, 5.0) | 4.0 (3.0, 5.0) |  |
| **Previous disease, n (%)** |  |  |  |  |
| Hypertension | 2,683 (79.4) | 778 (80.9) | 1,905 (78.7) | 0.160 |
| Coronary artery disease | 717 (21.2) | 193 (20.1) | 524 (21.6) | 0.337 |
| Congestive heart failure | 756 (22.4) | 258 (26.8) | 498 (20.6) | < 0.001 |
| Diabetes | 716 (21.2) | 233 (24.2) | 483 (20.0) | 0.006 |
| Thromboembolism | 690 (20.4) | 159 (16.5) | 531 (22.0) | < 0.001 |
| Previous bleeding | 230 (6.8) | 62 (6.4) | 168 (6.9) | 0.663 |
| COPD | 241 (7.1) | 172 (7.1) | 69 (7.2) | 0.998 |
| Cancer | 467 (13.9) | 335 (13.9) | 132 (13.8) | 0.967 |
| Dementia | 65 (1.9) | 44 (1.8) | 21(2.2) | 0.591 |
| Multimorbidity | 2086 (61.7) | 1472 (60.8) | 614 (63.9) | 0.106 |
| **Pharmacotherapy, n (%)** |  |  |  |  |
| Antiarrhythmic drugs | 679 (20.1) | 206 (21.4) | 473 (19.5) | 0.243 |
| ACEI | 983 (29.1) | 284 (29.6) | 699 (28.9) | 0.739 |
| ARB | 950 (28.1) | 279 (29.0) | 671 (27.7) | 0.472 |
| Beta-blocker | 2,097 (62.0) | 601 (62.5) | 1,496 (61.8) | 0.726 |
| Statin | 1,597 (47.2) | 444 (46.2) | 1,153 (47.6) | 0.471 |
| Insulin | 123 (3.6) | 46 (4.8) | 77 (3.2) | 0.032 |
| Oral hypoglycemic drugs | 452 (13.4) | 144 (15.0) | 308 (12.7) | 0.092 |
| Diuretic | 1,605 (47.5) | 502 (52.2) | 1,103 (45.6) | <0.001 |
| Digoxin | 301 (8.9) | 93 (9.7) | 208 (8.6) | 0.352 |
| Verapamil | 39 (1.2) | 8 (0.8) | 31 (1.3) | 0.356 |
| Diltiazem | 209 (6.2) | 47 (4.9) | 162 (6.7) | 0.059 |
| PPI | 1030 (30.5) | 312 (32.4) | 718 (30.0) | 0.121 |
| H2-receptor antagonists | 101 (3.0) | 26 (2.7) | 75 (3.1) | 0.621 |
| COX2 inhibitor | 25 (0.7) | 9 (0.9) | 16 (0.7) | 0.535 |
| SSRI | 151 (4.5) | 44 (4.6) | 107 (4.4) | 0.914 |
| Polypharmacy | 2155 (63.7) | 621 (64.6) | 1,534 (63.4) | 0.527 |

Continuous variables were presented with mean (SD) and median (IQR). Category variables were presented with frequency and percentage (%).

Abbreviations: AF, atrial fibrillation; VKA, vitamin K antagonists; NOACs, Non-vitamin K oral anticoagulants; COPD, chronic obstructive pulmonary disease; ACEI, angiotensin-converting enzyme inhibitor; ARB, angiotensin II receptor blocker; PPI, proton-pump inhibitor; SSRI, selective serotonin reuptake inhibitor; SD, standard deviation; IQR, interquartile range.

# Supplementary table S6. Clinical events between VKA and NOACs anticoagulant treatment groups in AF patients aged ≥ 80 years

|  | **VKA, n (%)** | **NOACs, n (%)** | ***P* value** |
| --- | --- | --- | --- |
| All cause death | **184 (19.1)** | **356 (14.7)** | **0.002** |
| CV death | **82 (8.5)** | **153 (6.3)** | **0.027** |
| MACE | **127 (13.2)** | **249 (10.3)** | **0.017** |
| TE | 64 (6.6) | 143 (5.9) | 0.458 |
| Major bleeding | **67 (7.0)** | **117 (4.8)** | **0.016** |
| Stroke | 45 (4.7) | 98 (4.0) | 0.465 |
| MI | 18 (1.9) | 65 (2.7) | 0.209 |

MACE is a composite included CV death, stroke and MI.

TE is a composite included transit ischemic attack, stroke and non-CNS arterial embolism.

Abbreviations: AF, atrial fibrillation; VKA, vitamin K antagonists; NOACs, Non-vitamin K oral anticoagulants; CV death, cardiovascular death; MACE, major adverse cardiovascular events; TE, thromboembolism; MI, myocardial fraction; CNS, central nervous system.

# Supplementary table S7. Clinical events between VKA and different NOAC anticoagulant treatment groups in AF patients aged ≥ 80 years

|  | **VKA**  **(N = 961)** | **Apixaban**  **(N = 1,050)** | **Dabigatran**  **(N = 611)** | **Edoxaban**  **(N = 51)** | **Rivaroxaban**  **(N = 708)** | ***p* value** |
| --- | --- | --- | --- | --- | --- | --- |
| All cause death, n (%) | **184 (19.1)** | **144 (13.7) *** | **80 (13.1) *** | **10 (19.6)** | **122 (17.2)** | **0.002** |
| CV death, n (%) | **82 (8.5)** | **55 (5.2) *** | **39 (6.4) *** | **5 (9.8)** | **54 (7.6)** | **0.032** |
| MACE, n (%) | **127 (13.2)** | **105 (10.0) *** | **61 (10.0) *** | **8 (15.7)** | **75 (10.6)** | **0.102** |
| TE, n (%) | 64 (6.6) | 60 (5.7) | 32 (5.2) | 4 (7.8) | 47 (6.6) | 0.647 |
| Major bleeding, n (%) | **67 (7.0)** | **45 (4.3) *** | **26 (4.2) *** | **0 (0.0)** | **46 (6.5)** | **<0.001** |
| Stroke, n (%) | 45 (4.7) | 38 (3.6) | 23 (3.7) | 4 (7.8) | 33 (4.7) | 0.387 |
| MI, n (%) | 18 (1.9) | 37 (3.5) | 11 (1.8) | 2 (3.9) | 15 (2.1) | 0.078 |

* Using Pearson’s chi-square test to compare the clinical events in the groups of apixaban/dabigatran with group of VKA, and the *p* value < 0.05.

MACE is a composite included CV death, stroke and MI.

TE is a composite included transit ischemic attack, stroke and non-CNS arterial embolism.

Abbreviations: AF, atrial fibrillation; VKA, vitamin K antagonists; NOACs, Non-vitamin K oral anticoagulants; CV death, cardiovascular death; MACE, major adverse cardiovascular events; TE, thromboembolism; MI, myocardial fraction; CNS: central nervous system.

# Supplementary table S8. HRs (95% CI) of clinical events in AF patients aged ≥ 80 years comparing different type of NOACs with VKA

| **Events** | **Model 1** | | **Model 2** | | **Model 3** | | **Model 4** | |
| --- | --- | --- | --- | --- | --- | --- | --- | --- |
|  | **HR** | **95% CI** | **HR** | **95% CI** | **HR** | **95% CI** | **HR** | **95% CI** |
| **All cause death** |  |  |  |  |  |  |  |  |
| Apixaban | **0.65** | **0.52, 0.93** | **0.62** | **0.49, 0.79** | **0.65** | **0.51, 0.84** | **0.64** | **0.49, 0.82** |
| Dabigatran | **0.65** | **0.49, 0.86** | **0.66** | **0.49, 0.88** | **0.68** | **0.50, 0.92** | **0.67** | **0.50, 0.91** |
| Edoxaban | 0.92 | 0.45, 1.87 | 0.68 | 0.30, 1.55 | 0.38 | 0.12, 1.24 | 0.38 | 1.17, 1.21 |
| Rivaroxaban | 0.87 | 0.68, 1.12 | 0.83 | 0.65, 1.08 | 0.89 | 0.68, 1.16 | 0.88 | 0.68, 1.15 |
| **CV death** |  |  |  |  |  |  |  |  |
| Apixaban | **0.54** | **0.37, 0.79** | **0.55** | **0.38, 0.81** | **0.59** | **0.40, 0.88** | **0.58** | **0.39, 0.86** |
| Dabigatran | 0.66 | 0.44, 1.01 | 0.67 | 0.44, 1.04 | 0.68 | 0.44, 1.07 | 0.58 | 0.43, 1.06 |
| Edoxaban | 1.03 | 0.37, 2.84 | 0.80 | 0.24, 2.61 | 0.62 | 0.15, 2.51 | 0.58 | 0.14, 2.35 |
| Rivaroxaban | 0.88 | 0.61, 1.27 | 0.86 | 0.58, 1.25 | 0.89 | 0.60, 1.33 | 0.89 | 0.60, 1.33 |
| **MACE** |  |  |  |  |  |  |  |  |
| Apixaban | **0.72** | **0.55, 0.95** | **0.71** | **0.53, 0.94** | **0.74** | **0.55, 0.98** | **0.72** | **0.54, 0.96** |
| Dabigatran | **0.72** | **0.52, 0.99** | **0.70** | **0.50, 0.98** | **0.71** | **0.50, 1.00** | **0.70** | **0.49, 0.99** |
| Edoxaban | 0.97 | 0.43, 2.21 | 0.82 | 0.33, 2.02 | 0.55 | 0.17, 1.74 | 0.54 | 0.17, 1.74 |
| Rivaroxaban | 0.77 | 0.57, 1.05 | 0.75 | 0.55, 1.02 | 0.78 | 0.56, 1.07 | 0.76 | 0.56, 1.05 |
| **TE** |  |  |  |  |  |  |  |  |
| Apixaban | 0.81 | 0.55, 1.19 | 0.80 | 0.54, 1.17 | 0.80 | 0.54, 1.19 | 0.80 | 0.54, 1.18 |
| Dabigatran | 0.89 | 0.58, 1.38 | 0.86 | 0.55, 1.34 | 0.81 | 0.51, 1.28 | 0.81 | 1.51, 1.28 |
| Edoxaban | 1.08 | 0.34, 3.44 | 0.78 | 0.19, 3.20 | 0.43 | 0.06, 3.10 | 0.41 | 0.06, 3.00 |
| Rivaroxaban | 1.07 | 0.72, 1.59 | 1.04 | 0.70, 1.55 | 1.09 | 0.72, 1.63 | 1.09 | 0.72, 1.63 |
| **Major bleeding** |  |  |  |  |  |  |  |  |
| Apixaban | **0.63** | **0.43, 0.94** | **0.58** | **0.39, 0.87** | **0.61** | **0.40, 0.92** | **0.61** | **0.39, 0.91** |
| Dabigatran | 0.55 | 0.33, 0.90 | 0.59 | 0.36, 0.97 | 0.63 | 0.38, 1.04 | 0.63 | 0.38, 1.05 |
| Edoxaban | 1.23 | 0.57, 1.34 | 1.18 | 0.51, 2.34 | 1.10 | 0.63, 2.37 | 1.08 | 0.72, 2 .29 |
| Rivaroxaban | 1.01 | 0.68, 1.47 | 0.92 | 0.52, 1.37 | 0.94 | 0.62, 1.41 | 0.95 | 0.63, 1.43 |
| **Stroke** |  |  |  |  |  |  |  |  |
| Apixaban | 0.71 | 0.45, 1.13 | 0.64 | 0.40, 1.04 | 0.66 | 0.40, 1.07 | 0.64 | 0.39, 1.05 |
| Dabigatran | 0.87 | 0.45, 1.13 | 0.77 | 0.46, 1.29 | 0.73 | 0.42, 1.25 | 0.72 | 0.42, 1.23 |
| Edoxaban | 1.35 | 0.52, 1.45 | 0.88 | 0.22, 0.36 | 0.46 | 0.67, 3.19 | 0.51 | 0.70, 3.69 |
| Rivaroxaban | 0.98 | 0.42, 4.32 | 0.93 | 0.57, 1.51 | 0.76 | 0.58, 1.57 | 0.94 | 0.58, 1.55 |
| **MI** |  |  |  |  |  |  |  |  |
| Apixaban | 1.72 | 0.96, 3.11 | 1.80 | 0.97, 3.27 | 1.72 | 0.89, 3.31 | 1.68 | 0.87, 3.25 |
| Dabigatran | 0.91 | 0.41, 1.98 | 0.94 | 0.42, 2.08 | 0.98 | 0.44, 2.18 | 0.97 | 0.43, 2.18 |
| Edoxaban | 2.17 | 0.50, 9.39 | 1.18 | 0.15, 9.28 | 1.22 | 0.16, 9.32 | 1.13 | 0.15, 8.71 |
| Rivaroxaban | 1.02 | 0.49, 2.11 | 1.04 | 0.50, 2.18 | 1.04 | 0.48, 2.22 | 1.02 | 0.47, 2.21 |

Model 1: Univariable model.

Model 2: Adjusted by age, sex, race, body mass index, systolic blood pressure, smoking/alcohol status.

Model 3: Adjusted by age, sex, race, body mass index, systolic blood pressure, smoking/alcohol status, type of AF, hypertension, coronary artery disease, chronic heart failure, diabetes, previous bleeding, TE, ACEI, COPD, cancer, dementia.

Model 4: age, sex, race, body mass index, systolic blood pressure, smoking/alcohol status, type of AF, hypertension, coronary artery disease, chronic heart failure, diabetes, previous bleeding, TE, COPD, cancer, dementia, ACEI, ARB, arrhythmic drugs, beta-blocker, statin.

MACE is a composite included CV death, stroke and MI.

TE is a composite included transit ischemic attack, stroke and non-CNS arterial embolism.

Abbreviations: AF, atrial fibrillation; VKA, vitamin K antagonists; NOACs, Non-vitamin K oral anticoagulants; HR, hazard ratio; 95% CI, 95% confidence interval; CV death, cardiovascular death; MACE, major adverse cardiovascular events; TE, thromboembolism; MI, myocardial fraction; CNS, central nervous system; BMI, body mass index; SBP, systolic blood pressure; ACEI, angiotensin-converting enzyme inhibitor; ARB, angiotensin II receptor blocker; COPD, chronic obstructive pulmonary disease.

# Supplementary table S9. Cox regression analysis of the association between clinical events and NOAC in AF patients aged ≥80 years across different regions

|  | **Europe (N = 1,981)** | **Asia (N = 277)** | **North America (N = 811)** | **Latin America (N =312)** |
| --- | --- | --- | --- | --- |
| **All cause death** |  |  |  |  |
| Crude model, HR (95% CI) | **0.77 (0.62, 0.97)** | 1.56 (0.54, 4.49) | 0.65 (0.44, 0.97) | 0.72 (0.43, 1.20) |
| Adjusted model, HR (95% CI) | **0.66 (0.51, 0.87)** | 1.43 (0.43, 4.93) | 0.78 (0.48, 0.26) | 0.66 (0.33, 1.30) |
| P for interaction | 0.704 | | | |
| **Cardiovascular death** |  |  |  |  |
| Crude model, HR (95% CI) | **0.60 (0.43, 0.85)** | 3.59 (0.48, 27.03) | 0.88 (0.47, 1.68) | 0.88 (0.42, 1.80) |
| Adjusted model, HR (95% CI) | **0.45 (0.29, 0.68)** | 3.38 (0.34, 33.26) | 1.50 (0.64, 3.53) | 1.12 (0.37, 3.37) |
| P for interaction | **0.017** | | | |
| **TE** |  |  |  |  |
| Crude model, HR (95% CI) | 0.80 (0.55, 1.18) | 2.55 (0.66, 12.4) | 0.68 (0.34, 1.41) | 1.65 (0.45, 6.12) |
| Adjusted model, HR (95% CI) | 0.68 (0.45, 1.03) | 3.20 (0.65, 15.5) | 0.77 (0.36, 1.65) | 3.22 (0.49, 25.55) |
| P for interaction | 0.267 | | | |
| **MACE** |  |  |  |  |
| Crude model, HR (95% CI) | **0.36 (0.19, 0.67)** | 3.47 (0.83, 14.6) | 0.87 (0.51, 1.49) | 1.18 (0.51, 2.32) |
| Adjusted model, HR (95% CI) | **0.57 (0.41, 0.79)** | 3.83 (0.86, 16.6) | 0.98 (0.56, 1.72) | 1.44 (0.61, 3.43) |
| P for interaction | 0.096 | | | |
| **Major Bleeding** |  |  |  |  |
| Crude model, HR (95% CI) | **0.47 (0.31, 0.72)** | 1.68 (0.20, 13.83) | 1.31 (0.64, 2.67) | 0.58 (0.17, 2.01) |
| Adjusted model, HR (95% CI) | **0.44 (0.28, 0.71)** | 1.81 (0.12, 6.35) | 1.46 (0.65, 3.29) | 0.47 (0.11, 2.11) |
| P for interaction | 0.707 | | | |
| **Stroke** |  |  |  |  |
| Crude model, HR (95% CI) | 0.68 (0.42, 0.95) | 1.98 (0.47, 8.29) | 0.62 (0.28, 1.41) | 2.39 (0.51, 11.29) |
| Adjusted model, HR (95% CI) | 0.53 (0.32, 0.90) | 2.03 (0.39, 1.05) | 0.76 (0.31, 1.29) | 4.01 (0.64, 2.52) |
| P for interaction | 0.162 | | | |
| **MI** |  |  |  |  |
| Crude model, HR (95% CI) | 1.27 (0.59, 2.73) | 1.69 (0.20, 14.02) | 0.92 (0.34, 2.47) | 1.62 (0.33, 7.86) |
| Adjusted model, HR (95% CI) | 1.14 (0.45, 2.91) | 1.02 (0.16, 6.53) | 0.97 (0.34, 2.81) | 4.11 (0.66, 2.56) |
| P for interaction | 0.801 | | | |

Adjusted for age, sex, race, body mass index, systolic blood pressure, smoking/alcohol status, type of AF, hypertension, coronary artery disease, chronic heart failure, diabetes, previous bleeding, TE, COPD, cancer, dementia, oral anticoagulants, ACEI, ARB, arrhythmic drugs, beta-blocker, statin.

MACE is a composite included CV death, stroke and MI.

TE is a composite included transit ischemic attack, stroke and non-CNS arterial embolism.

Abbreviations: AF, atrial fibrillation; VKA, vitamin K antagonists; NOACs, Non-vitamin K oral anticoagulants; HR, hazard ratio; 95% CI, 95% confidence interval; CV death, cardiovascular death; MACE, major adverse cardiovascular events; TE, thromboembolism; MI, myocardial fraction; CNS, central nervous system; ACEI, angiotensin-converting enzyme inhibitor; ARB, angiotensin II receptor blocker; COPD, chronic obstructive pulmonary disease.

# Supplementary table S10. Cox regression analysis of the association between clinical events and NOAC in AF patients aged ≥80 years across different race and ethnicity

|  | **White (N= 2,604)** | **Asian (N = 603)** | **Others (N = 174)** |
| --- | --- | --- | --- |
| **All cause death** |  |  |  |
| Crude model, HR (95% CI) | **0.73 (0.59, 0.91)** | 1.61 (0.56, 4.59) | 0.51 (0.31, 0.84) |
| Adjusted model, HR (95% CI) | **0.69 (0.55, 0.87)** | 1.54 (0.47, 5.04) | 0.55 (0.29, 1.05) |
| P for interaction | 0.983 | | |
| **Cardiovascular death** |  |  |  |
| Crude model | **0.67 (0.48, 0.93)** | 3.32 (0.45, 25.15) | 0.47 (0.24, 0.94) |
| Adjusted model | **0.66 (0.47, 0.97)** | 3.75 (0.33, 4.22) | 0.39 (0.14, 1.12) |
| P for interaction | 0.845 | | |
| **TE** |  |  |  |
| Crude model, HR (95% CI) | 0.88 (0.61, 1.25) | 2.59 (0.60, 11.23) | 0.58 (0.27, 1.23) |
| Adjusted model, HR (95% CI) | 0.80 (0.54, 1.16) | 2.80 (0.56, 13.91) | 0.54 (0.24, 1.26) |
| P for interaction | 0.703 | | |
| **MACE** |  |  |  |
| Crude model, HR (95% CI) | **0.83 (0.63, 0.96)** | 3.42 (0.81, 14.41) | 1.02 (0.41, 2.51) |
| Adjusted model HR (95% CI) | **0.70 (0.53, 0.93)** | 4.15 (0.96, 18.06) | 1.33 (0.21, 8.41) |
| P for interaction | 0.353 | | |
| **Major Bleeding** |  |  |  |
| Crude model, HR (95% CI) | **0.67 (0.48, 0.95)** | 0.92 (0.19, 4.40) | 1.36 (0.39, 4.73) |
| Adjusted model HR (95% CI) | **0.64 (0.45, 0.93)** | 0.32 (0.10, 2.34) | 2.20 (0.31, 15.65) |
| P for interaction | 0.207 | | |
| **Stroke** |  |  |  |
| Crude model, HR (95% CI) | 0.82 (0.53, 1.28) | 1.81 (0.42, 7.27) | 0.50 (0.22, 1.13) |
| Adjusted model HR (95% CI) | 0.71 (0.44, 1.14) | 1.61 (0.32, 8.12) | 0.42 (0.17, 1.07) |
| P for interaction | 0.495 | | |
| **Myocardial fraction** |  |  |  |
| Crude model | 1.37 (0.75, 2.51) | 1.97 (0.24, 16.19) | 0.70 (0.14, 3.59) |
| HR (95% CI) | 1.18 (0.59, 2.32) | 1.95 (0.20, 18.90) | 0.24 (0.02, 5.58) |
| P for interaction | 0.867 | | |
| Adjusted for age, sex, race, body mass index, systolic blood pressure, smoking/alcohol status, type of AF, hypertension, coronary artery disease, chronic heart failure, diabetes, previous bleeding, TE, COPD, cancer, dementia, oral anticoagulants, ACEI, ARB, arrhythmic drugs, beta-blocker, statin.  MACE is a composite included CV death, stroke and MI.  TE is a composite included transit ischemic attack, stroke and non-CNS arterial embolism.  Abbreviations: AF, atrial fibrillation; VKA, vitamin K antagonists; NOACs, Non-vitamin K oral anticoagulants; HR, hazard ratio; 95% CI, 95% confidence interval; CV death, cardiovascular death; MACE, major adverse cardiovascular events; TE, thromboembolism; MI, myocardial fraction; CNS, central nervous system; ACEI, angiotensin-converting enzyme inhibitor; ARB, angiotensin II receptor blocker; COPD, chronic obstructive pulmonary disease. | | | |

# Supplementary table S11. Subgroup analysis of the association between clinical events and NOACs in AF patients aged ≥ 80 years

|  | **Subgroups** | **HR (95% CI)** | ***p* value** | ***p* for interaction** |
| --- | --- | --- | --- | --- |
| **All cause death** | **Sex** |  |  | 0.182 |
|  | Male (n=1,479) | 0.62 (0.46, 0.82) | 0.001 |  |
|  | Female (n=1,902) | 0.84 (0.60, 0.96) | 0.026 |  |
|  | **Type of AF** |  |  | 0.720 |
|  | Paroxysmal AF (n=1,701) | 0.76 (0.54, 0.98) | 0.042 |  |
|  | Non- Paroxysmal AF (n=1,680) | 0.69 (0.53, 0.90) | 0.006 |  |
| **Cardiovascular death** | **Sex** |  |  | 0.271 |
|  | Male (n=1,479) | 0.59 (0.37, 0.93) | 0.024 |  |
|  | Female (n=1,902) | 0.89 (0.54, 1.43) | 0.631 |  |
|  | **Type of AF** |  |  | 0.143 |
|  | Paroxysmal AF (n=1,701) | 0.55 (0.32, 0.94) | 0.029 |  |
|  | Non- Paroxysmal AF (n=1,680) | 0.87 (0.57, 1.34) | 0.554 |  |
| **TE** | **Sex** |  |  | 0.542 |
|  | Male (n=1,479) | 0.65 (0.40, 1.05) | 0.079 |  |
|  | Female (n=1,902) | 1.03 (0.77, 1.68) | 0.389 |  |
|  | **Type of AF** |  |  | 0.208 |
|  | Paroxysmal AF (n=1,701) | 0.57 (0.34, 0.98) | 0.045 |  |
|  | Non- Paroxysmal AF (n=1,680) | 1.03 (0.67, 1.59) | 0.883 |  |
| **MACE** | **Sex** |  |  | 0.019 |
|  | Male (n=1,479) | 0.54 (0.38, 0.77) | <0.001 |  |
|  | Female (n=1,902) | 0.98 (0.68, 1.42) | 0.956 |  |
|  | **Type of AF** |  |  | 0.223 |
|  | Paroxysmal AF (n=1,701) | 0.58 (0.39, 0.86) | 0.008 |  |
|  | Non- Paroxysmal AF (n=1,680) | 0.84 (0.63, 1.13) | 0.245 |  |
| **Major bleeding** | **Sex** |  |  | 0.841 |
|  | Male (n=1,479) | 0.64 (0.42, 0.98) | 0.047 |  |
|  | Female (n=1,902) | 0.72 (0.45, 1.18) | 0.198 |  |
|  | **Type of AF** |  |  | 0.149 |
|  | Paroxysmal AF (n=1,701) | 0.55 (0.32, 0.94) | 0.029 |  |
|  | Non- Paroxysmal AF (n=1,680) | 0.84 (0.54, 0.97) | 0.046 |  |
| **Stroke** | **Sex** |  |  | 0.078 |
|  | Male (n=1,479) | 0.53 (0.28, 0.93) | 0.028 |  |
|  | Female (n=1,902) | 1.03 (0.59, 1.87) | 0.845 |  |
|  | **Type of AF** |  |  | 0.472 |
|  | Paroxysmal AF (n=1,701) | 0.54 (0.28, 1.04) | 0.064 |  |
|  | Non- Paroxysmal AF (n=1,680) | 0.83 (0.50, 1.37) | 0.471 |  |
| **MI** | **Sex** |  |  | 0.166 |
|  | Male (n=1,479) | 1.08 (0.43, 1.92) | 0.619 |  |
|  | Female (n=1,902) | 1.98 (0.75, 5.21) | 0.165 |  |
|  | **Type of AF** |  |  | 0.522 |
|  | Paroxysmal AF (n=1,701) | 0.86 (0.25, 2.92) | 0.809 |  |
|  | Non- Paroxysmal AF (n=1,680) | 1.43 (0.68, 3.02) | 0.345 |  |

Model were adjusted by age, sex, race, BMI, SBP, smoking/alcohol status, type of AF, hypertension, coronary artery disease, chronic heart failure, diabetes, previous bleeding, TE, COPD, cancer, dementia, oral anticoagulation, ACEI, ARB, arrhythmic drugs, beta-blocker, statin.

MACE is a composite included CV death, stroke and MI.

TE is a composite included transit ischemic attack, stroke and non-CNS arterial embolism.

Abbreviations: AF, atrial fibrillation; HR, hazard ratio; 95% CI, 95% confidence interval; CV death, cardiovascular death; MACE, major adverse cardiovascular events; TE, thromboembolism; MI, myocardial fraction; CNS, central nervous system; BMI, body mass index; SBP, systolic blood pressure; ACEI, angiotensin-converting enzyme inhibitor; ARB, angiotensin II receptor blocker; COPD, chronic obstructive pulmonary disease.

# Supplementary table S12. The association between clinical events and aged ≥80 years in frail AF patients

|  | **Subgroups** | **HR (95% CI)** | ***p* value** | ***p* for interaction** |
| --- | --- | --- | --- | --- |
| **All cause death** | **BMI** |  |  | 0.804 |
|  | ≤23 kg/m^2^ (n=1,479) | 1.92 (1.13, 3.28) | 0.016 |  |
|  | >23 kg/m^2^ (n=6,173) | 2.03 (1.71, 2.41) | <0.001 |  |
|  | **Multimorbidity** |  |  | 0.175 |
|  | Yes (n=4,579) | 2.02 (1.55, 2.64) | <0.001 |  |
|  | No (n=3,054) | 2.74 (2.00, 3.77) | <0.001 |  |
|  | **Polypharmacy** |  |  | 0.058 |
|  | Yes (n=4,594) | 1.85 (1.54, 2.22) | <0.001 |  |
|  | No (n=3,058) | 2.39 (1.82, 3.13) | <0.001 |  |
| **Cardiovascular death** | **BMI** |  |  | 0.271 |
|  | ≤23 kg/m^2^ (n=1,479) | 1.75 (1.26, 2.44) | <0.001 |  |
|  | >23 kg/m^2^ (n=6,173) | 2.28 (1.76, 2.96) | <0.001 |  |
|  | **Multimorbidity** |  |  | 0.058 |
|  | Yes (n=4,579) | 1.79 (1.51, 2.13) | <0.001 |  |
|  | No (n=3,054) | 3.23 (2.00, 5.24) | <0.001 |  |
|  | **Polypharmacy** |  |  | 0.129 |
|  | Yes (n=4,594) | 2.01 (1.52,2.62) | <0.002 |  |
|  | No (n=3,058) | 2.95 (1.90, 4.59) | <0.001 |  |
| **TE** | **BMI** |  |  | 0.671 |
|  | ≤23 kg/m^2^ (n=1,479) | 1.68 (1.08, 2.62) | 0.021 |  |
|  | >23 kg/m^2^ (n=6,173) | 1.39 (1.08,1.81) | 0.011 |  |
|  | **Multimorbidity** |  |  | 0.856 |
|  | Yes (n=4,579) | 1.51 (1.14, 2.00) | 0.003 |  |
|  | No (n=3,054) | 1.45 (0.97, 2.17) | 0.069 |  |
|  | **Polypharmacy** |  |  | 0.398 |
|  | Yes (n=4,594) | 1.38 (1.04, 1.86) | 0.029 |  |
|  | No (n=3,058) | 1.75 (1.22, 2.52) | 0.002 |  |
| **MACE** | **BMI** |  |  | 0.680 |
|  | ≤23 kg/m^2^ (n=1,479) | 1.64 (1.09, 2.46) | 0.016 |  |
|  | >23 kg/m^2^ (n=6,173) | 1.55 (1.29, 1.88) | <0.001 |  |
|  | **Multimorbidity** |  |  | 0.290 |
|  | Yes (n=4,579) | 1.51 (1.24, 1.85) | <0.001 |  |
|  | No (n=3,054) | 1.89 (1.36, 2.61) | <0.001 |  |
|  | **Polypharmacy** |  |  | 0.151 |
|  | Yes (n=4,594) | 2.01 (1.52, 2.65) | <0.001 |  |
|  | No (n=3,058) | 1.94 (1.43, 2.63) | <0.001 |  |
| **Major bleeding** | **BMI** |  |  | 0.226 |
|  | ≤23 kg/m^2^ (n=1,479) | 1.62 (0.93, 2.83) | 0.091 |  |
|  | >23 kg/m^2^ (n=6,173) | 1.21 (0.93, 1.55) | 0.160 |  |
|  | **Multimorbidity** |  |  | 0.208 |
|  | Yes (n=4,579) | 0.36 (0.88, 1.42) | 0.358 |  |
|  | No (n=3,054) | 1.21 (0.93, 1.55) | 0.160 |  |
|  | **Polypharmacy** |  |  | 0.084 |
|  | Yes (n=4,594) | 1.81 (1.19, 2.76) | 0.006 |  |
|  | No (n=3,058) | 1.13 (0.86, 1.50) | 0.378 |  |
| **Stroke** | **BMI** |  |  | 0.475 |
|  | ≤23 kg/m^2^ (n=1,479) | 1.68 (1.02, 2.81) | 0.043 |  |
|  | >23 kg/m^2^ (n=6,173) | 1.28 (0.74, 1.75) | 0.121 |  |
|  | **Multimorbidity** |  |  | 0.916 |
|  | Yes (n=4,579) | 1.58 (1.18, 2.12) | 0.002 |  |
|  | No (n=3,054) | 1.41 (0.88, 2.26) | 0.155 |  |
|  | **Polypharmacy** |  |  | 0.337 |
|  | Yes (n=4,594) | 1.75 (1.13, 2.71) | 0.011 |  |
|  | No (n=3,058) | 1.24 (0.86, 1.79) | 0.238 |  |
| **MI** | **BMI** |  |  | 0.846 |
|  | ≤23 kg/m^2^ (n=1,479) | 1.98 (0.94, 5.32) | 0.176 |  |
|  | >23 kg/m^2^ (n=6,173) | 1.48 (1.04, 2.13) | 0.034 |  |
|  | **Multimorbidity** |  |  | 0.136 |
|  | Yes (n=4,579) | 1.36 (0.94, 1.99) | 0.101 |  |
|  | No (n=3,054) | 2.52 (1.21, 5.26) | 0.013 |  |
|  | **Polypharmacy** |  |  | 0.168 |
|  | Yes (n=4,594) | 1.37 (0.94, 2.01) | 0.101 |  |
|  | No (n=3,058) | 2.41 (1.14, 5.09) | 0.021 |  |

Model was adjusted by age, sex, race, body mass index, systolic blood pressure, smoking/alcohol status, type of AF, hypertension, coronary artery disease, chronic heart failure, diabetes, previous bleeding, TE, COPD, cancer, dementia, oral anticoagulation, ACEI, ARB, arrhythmic drugs, beta-blocker, statin.

MACE is a composite included CV death, stroke and MI.

TE is a composite included transit ischemic attack, stroke and non-CNS arterial embolism.

Multimorbidity was defined as the presence of more than two comorbidities other than atrial fibrillation.

Polypharmacy was defined as the use of five or more prescription drugs.

Abbreviations: AF, atrial fibrillation; HR, hazard ratio; 95% CI, 95% confidence interval; CV death, cardiovascular death; MACE, major adverse cardiovascular events; TE, thromboembolism; MI, myocardial fraction; CNS, central nervous system; ACEI, angiotensin-converting enzyme inhibitor; ARB, angiotensin II receptor blocker; COPD, chronic obstructive pulmonary disease

# Supplementary table S13. The association between clinical events and NOACs in frail AF patients aged ≥ 80 years

|  | **Subgroups** | **HR (95% CI)** | ***p* value** | ***p* for interaction** |
| --- | --- | --- | --- | --- |
| **All cause death** | **BMI** |  |  | 0.352 |
|  | ≤23 kg/m^2^ (n=701) | 0.61 (0.42, 0.88) | 0.007 |  |
|  | >23 kg/m^2^ (n=2,680) | 0.80 (0.65, 0.98) | 0.029 |  |
|  | **multimorbidity** |  |  | 0.272 |
|  | Yes (n=2,086) | 0.69 (0.48, 0.99) | 0.046 |  |
|  | No (n=1,285) | 0.61 (0.43, 0.87) | 0.006 |  |
|  | **Polypharmacy** |  |  | 0.677 |
|  | Yes (n=2,155) | 0.74 (0.60, 0.93) | 0.007 |  |
|  | No (n=1,226) | 0.91 (0.62, 1.33) | 0.639 |  |
| **Cardiovascular death** | **BMI** |  |  | 0.719 |
|  | ≤23 kg/m^2^ (n=701) | 0.77 (0.63, 0.95) | 0.013 |  |
|  | >23 kg/m^2^ (n=2,680) | 0.60 (0.34, 1.05) | 0.074 |  |
|  | **multimorbidity** |  |  | 0.576 |
|  | Yes (n=2,086) | 0.58 (0.34, 0.98) | 0.041 |  |
|  | No (n=1,285) | 0.43 (0.22, 0.87) | 0.019 |  |
|  | **Polypharmacy** |  |  | 0.758 |
|  | Yes (n=2,155) | 0.71 (0.52, 0.97) | 0.040 |  |
|  | No (n=1,226) | 0.76 (0.46, 1.25) | 0.274 |  |
| **TE** | **BMI** |  |  | 0461 |
|  | ≤23 kg/m^2^ (n=701) | 0.86 (0.33, 2.23) | 0.751 |  |
|  | >23 kg/m^2^ (n=2,680) | 0.88 (0.63, 1.21) | 0.409 |  |
|  | **multimorbidity** |  |  | 0.901 |
|  | Yes (n=2,086) | 0.98 (0.69, 1.40) | 0.930 |  |
|  | No (n=1,285) | 0.81 (0.47, 1.37) | 0.429 |  |
|  | **Polypharmacy** |  |  | 0.152 |
|  | Yes (n=2,155) | 0.78 (0.54, 1.12) | 0.185 |  |
|  | No (n=1,226) | 0.92 (0.48, 1.75) | 0.792 |  |
| **MACE** | **BMI** |  |  | 0.645 |
|  | ≤23 kg/m^2^ (n=701) | 0.72 (0.56, 0.91) | 0.006 |  |
|  | >23 kg/m^2^ (n=2,680) | 0.84 (0.52, 1.35) | 0.481 |  |
|  | **multimorbidity** |  |  | 0.104 |
|  | Yes (n=2,086) | 0.61 (0.40, 0.94) | 0.024 |  |
|  | No (n=1,285) | 0.87 (0.68, 1.14) | 0.329 |  |
|  | **Polypharmacy** |  |  | 0.216 |
|  | Yes (n=2,155) | 0.62 (0.48, 0.79) | <0.001 |  |
|  | No (n=1,226) | 0.35 (0.26, 0.47) | <0.001 |  |
| **Major bleeding** | **BMI** |  |  | 0.708 |
|  | ≤23 kg/m^2^ (n=701) | 0.51 (0.29, 0.88) | 0.017 |  |
|  | >23 kg/m^2^ (n=2,680) | 0.56 (0.41, 0.78) | <0.001 |  |
|  | **multimorbidity** |  |  | 0.201 |
|  | Yes (n=2,086) | 0.62 (0.42, 0.91) | 0.014 |  |
|  | No (n=1,285) | 0.39 (0.23, 0.66) | <0.001 |  |
|  | **Polypharmacy** |  |  | 0.512 |
|  | Yes (n=2,155) | 0.61 (0.41, 0.92) | 0.019 |  |
|  | No (n=1,226) | 0.71 (0.42, 1.21) | 0.207 |  |
| **Stroke** | **BMI** |  |  | 0.352 |
|  | ≤23 kg/m^2^ (n=701) | 1.21 (0.55, 2.68) | 0.629 |  |
|  | >23 kg/m^2^ (n=2,680) | 0.76 (0.51, 1.13) | 0.176 |  |
|  | **multimorbidity** |  |  | 0.732 |
|  | Yes (n=2,086) | 0.78 (0.46, 1.30) | 0.338 |  |
|  | No (n=1,285) | 0.94 (0.44, 2.04) | 0.878 |  |
|  | **Polypharmacy** |  |  | 0.603 |
|  | Yes (n=2,155) | 0.81 (0.51, 1.26) | 0.337 |  |
|  | No (n=1,226) | 0.91 (0.52, 1.62) | 0.763 |  |
| **MI** | **BMI** |  |  | 0.829 |
|  | ≤23 kg/m^2^ (n=701) | 1.99 (0.44, 9.04) | 0.369 |  |
|  | >23 kg/m^2^ (n=2,680) | 1.35 (0.77, 2.36) | 0.289 |  |
|  | **multimorbidity** |  |  | 0.431 |
|  | Yes (n=2,086) | 1.23 (0.62, 2.41) | 0.555 |  |
|  | No (n=1,285) | 2.13 (0.64, 7.07) | 0.216 |  |
|  | **Polypharmacy** |  |  | 0.606 |
|  | Yes (n=2,155) | 1.17 (0.61, 2.27) | 0.642 |  |
|  | No (n=1,226) | 1.37 (0.51, 3.71) | 0.526 |  |

Model were adjusted by age, sex, race, BMI, SBP, smoking/alcohol status, type of AF, hypertension, coronary artery disease, chronic heart failure, diabetes, previous bleeding, TE, COPD, cancer, dementia, oral anticoagulation, ACEI, ARB, arrhythmic drugs, beta-blocker, statin.

MACE is a composite included CV death, stroke and MI.

TE is a composite included transit ischemic attack, stroke and non-CNS arterial embolism.

Multimorbidity was defined as the presence of more than two comorbidities other than atrial fibrillation.

Polypharmacy was defined as the use of five or more prescription drugs.

Abbreviations: AF, atrial fibrillation; HR, hazard ratio; 95% CI, 95% confidence interval; CV death, cardiovascular death; MACE, major adverse cardiovascular events; TE, thromboembolism; MI, myocardial fraction; CNS, central nervous system; BMI, body mass index; SBP, systolic blood pressure; ACEI, angiotensin-converting enzyme inhibitor; ARB, angiotensin II receptor blocker; COPD, chronic obstructive pulmonary disease.

# Supplementary Figure S1. Forest plot of clinical events in AF patients by Cox regression analysis comparing group of aged 75-79 years (reference) and aged ≥80 years


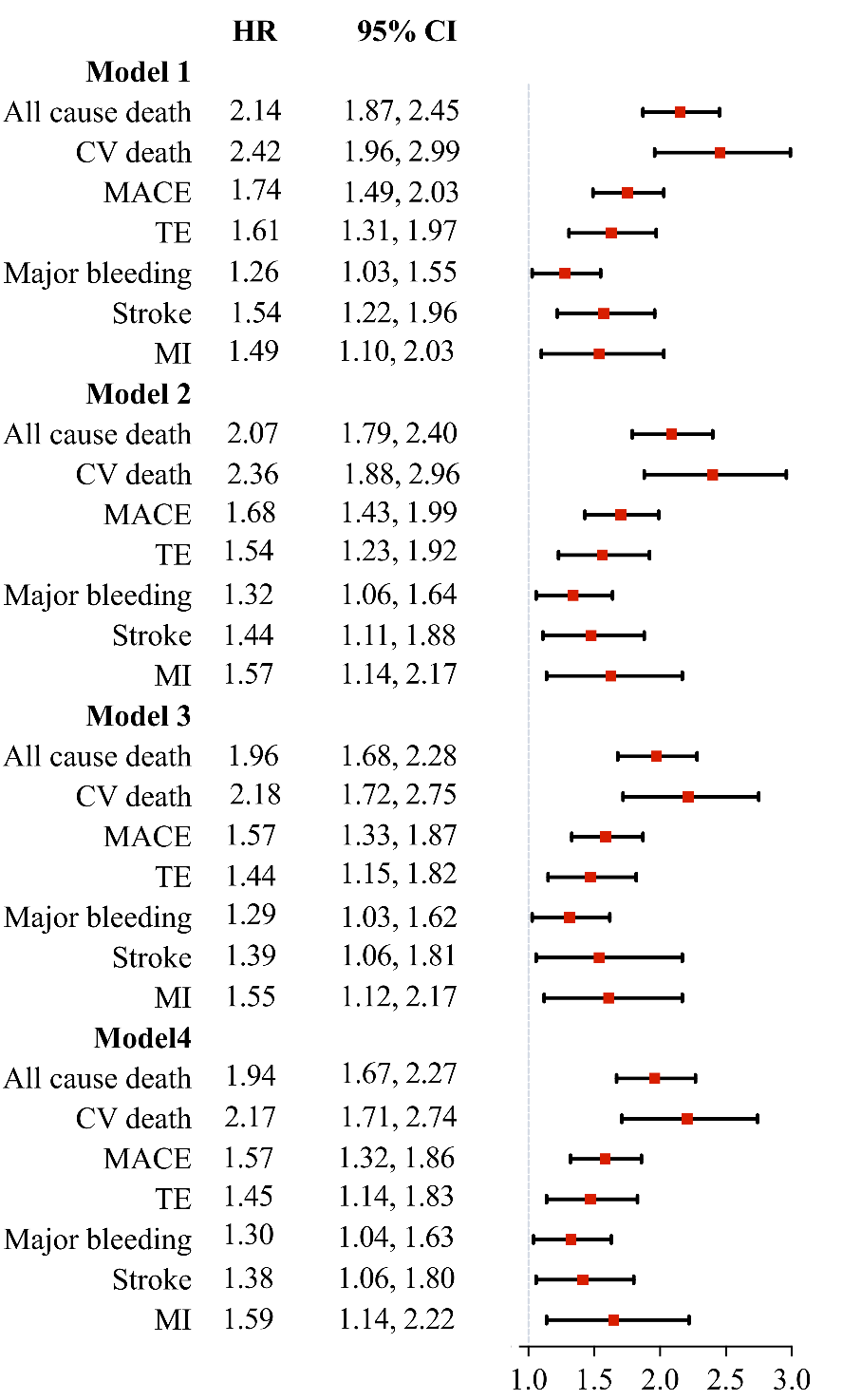


Model 1: Univariable model. Model 2: Adjusted by age, sex, race, body mass index, systolic blood pressure, smoking/alcohol status. Model 3: Adjusted by age, sex, race, body mass index, systolic blood pressure, smoking/alcohol status, type of AF, hypertension, coronary artery disease, chronic heart failure, diabetes, previous bleeding, TE, COPD, cancer, dementia. Model 4: age, sex, race, body mass index, systolic blood pressure, smoking/alcohol status, type of AF, hypertension, coronary artery disease, chronic heart failure, diabetes, previous bleeding, TE, COPD, cancer, dementia, oral anticoagulants, ACEI, ARB, arrhythmic drugs, beta-blocker, statin.

MACE is a composite included CV death, stroke and MI. TE is a composite included transit ischemic attack, stroke and non-CNS arterial embolism.

Abbreviations: AF, atrial fibrillation; HR, hazard ratio; 95% CI, 95% confidence interval; CV death, cardiovascular death; MACE, major adverse cardiovascular events; TE, thromboembolism; MI, myocardial fraction; CNS, central nervous system; ACEI, angiotensin-converting enzyme inhibitor; ARB, angiotensin II receptor blocker; COPD, chronic obstructive pulmonary disease.

# Supplementary Figure S2. Cumulative event curve in very old AF patients treated with NOACs and VKA.


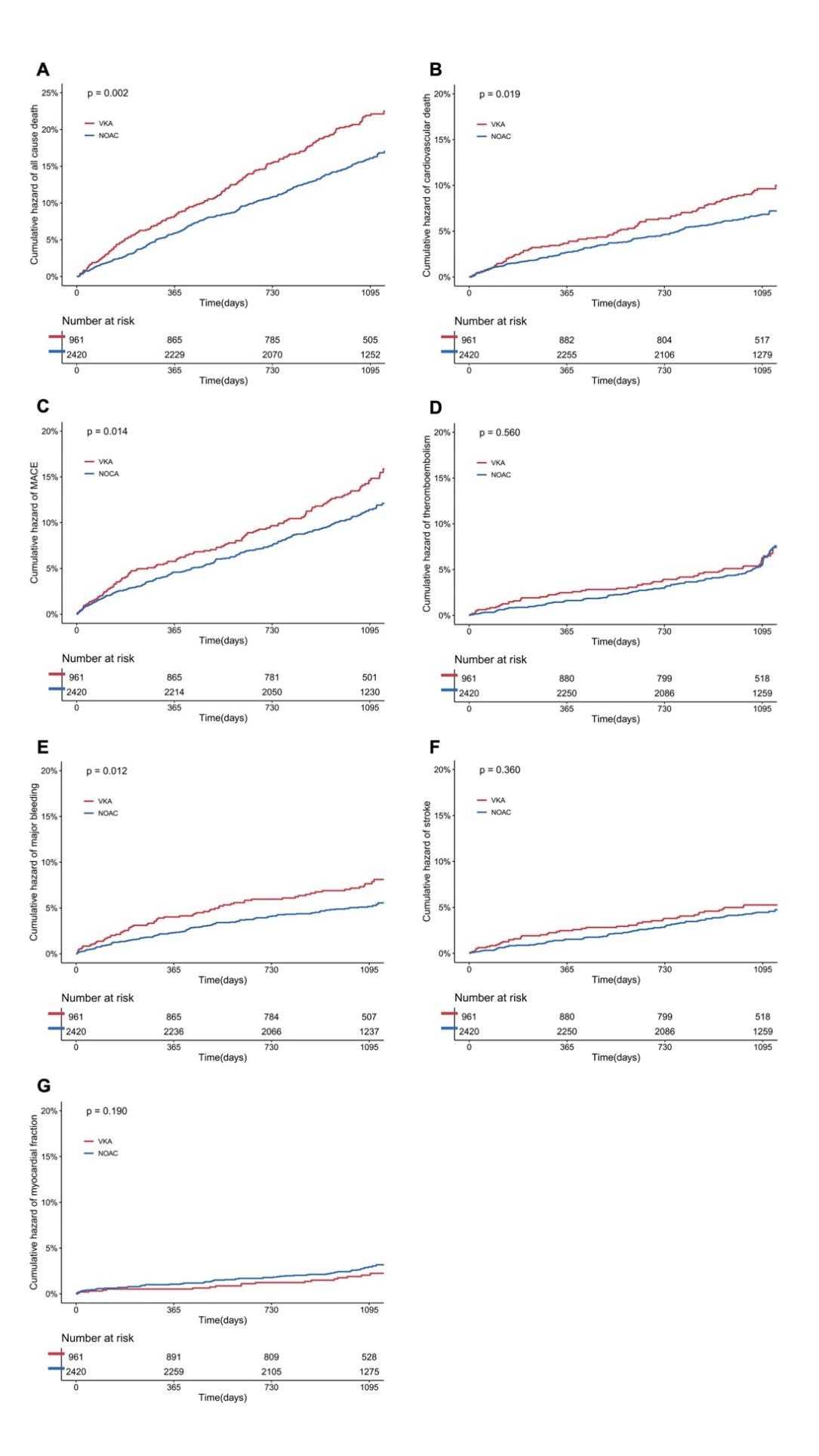


A-G were all cause death, cardiovascular death, MACE, thromboembolism, major bleeding, stroke and myocardial fraction, respectively. Abbreviation: MACE, major adverse cardiovascular events.
